# Sensory cells and the organization of the peripheral nervous system of the siboglinid *Oligobrachia haakonmosbiensis* Smirnov, 2000

**DOI:** 10.1186/s40850-022-00114-z

**Published:** 2022-03-29

**Authors:** Olga V. Zaitseva, Roman V. Smirnov, Zinaida I. Starunova, Andrey A. Vedenin, Viktor V. Starunov

**Affiliations:** 1 Zoological Institute, Russian Academy of Sciences, 1 Universitetskaya Emb, St Petersburg, 199034 Russia; 2 P.P. Shirshov Institute of Oceanology, Russian Academy of Sciences, 36 Nakhimovskiy Ave, Moscow, 117997 Russia; 3 Saint-Petersburg State University 7-9 Universitetskaya Emb, St Petersburg, 199034 Russia

**Keywords:** Morphology, Peripheral nervous system, Receptors, Pogonophora, Siboglinidae, Immunohistochemistry, CLSM, SEM

## Abstract

**Background:**

The nervous system of siboglinids has been studied mainly in *Osedax* and some Vestimentifera, while data in Frenulata – one of the four pogonophoran main branches – is still fragmentary. In most of the studies, the focus is almost always on the central nervous system, while the peripheral nervous system has traditionally received little attention. In contrast to other annelids, the structure and diversity of sensory structures in siboglinids are still quite undescribed. Meanwhile, the peripheral nervous system, as well as sensory elements, are extremely evolutionarily labile, and information about their organization is of high importance to understand lifestyles and behavior as well as main trends that lead siboglinids to their peculiar organization.

**Results:**

The structure of the peripheric nervous system, sensory elements, and neuromuscular relationships of *Oligobrachia haakonmosbiensis* were studied using both scanning electron and confocal laser microscopy. A significant number of monociliary sensory cells, as well as sensory complexes located diffusely in the epithelium of the whole body were revealed. The latter include the cephalic tentacles, sensory cells accumulations along the dorsal furrow and ciliary band, areas of the openings of the tubiparous glands, and papillae. The oval ciliary spot located on the cephalic lobe at the base of the tentacles can also be regarded as a sensory organ. Most of the detected sensory cells show immunoreactivity to substance P and/or acetylated α-tubulin. FMRFamide- and serotonin-like immunoreactivity are manifested by neurons that mainly innervate tentacles, muscles, body wall epithelium, skin glands, tubiparous glands, and papillae. In the larva of *O. haakonmosbiensis*, monociliary sensory elements were revealed in the region of the apical organ, along the body, and on the pygidium.

**Conclusions:**

The diversity of sensory structures in *O. haakonmosbiensis* comprises epidermal solitary sensory cells, sensory spots around tubiparous glands openings, and putative sensory organs such as cephalic tentacles, an oval ciliary spot on the cephalic lobe, the dorsal furrow, and papillae. Sensory structures associated with papillae and tubiparous glands play presumable mechanosensory functions and are associated with regulation of tube building as well as anchorage of the worm inside the tube. Sensory structures of the dorsal furrow are presumably engaged in the regulation of reproductive behavior. An overall low level of morphological differentiation of *O. haakonmosbiensis* peripheral nervous system is not typical even for annelids with the intraepithelial nervous system. This can be considered as a plesiomorphic feature of its peripheral plexus’s organization, or as evidence for the neotenic origin of Siboglinidae.

**Supplementary Information:**

The online version contains supplementary material available at 10.1186/s40850-022-00114-z.

## Background

Siboglinids (Annelida: Siboglinidae a.k.a. Pogonophora) is a group of enigmatic tube-dwelling annelids with highly specialized physiological and morphological traits. All four main branches of siboglinids (giant vestimentiferans, small classical pogonophorans – frenulates, “woodworms” moniliferans, and “bone-eating” *Osedax*) are currently united in the Siboglinidae clade and placed within Sedentaria as sister to Cirratulida [1–14]. Investigating the structure of the key organ systems, such as musculature, coeloms, nervous system, blood vessels etc., provides material for a comprehensive comparative morphological analysis, that, together with a molecular phylogenetic essay may give new insights about main evolutionary trends in this extraordinary annelid clade. In this respect, the nervous system seems to be one of the most promising, since it is well studied in a large number of annelid representatives and combines both conservative and highly plastic features [15–18]. However, data on anatomical organization of the nervous system in frenulates and other siboglinids remains hitherto fragmentary.

The nervous system is traditionally divided into central and peripheral parts. In Annelids the first one comprises the brain and ventral nerve cords. The latter is composed of transversal and longitudinal nerves of the body wall, the stomatogastric nervous system (lacking in all pogonophorans), and peripheral nerve centers, such as parapodial or pharyngeal ganglia [15, 19]. Among pogonophorans, the anatomy of the brain and other parts of the central nervous system (CNS) of *Osedax* and several vestimentiferans has been studied in the greatest detail using light microscopy, histological techniques [20–23], as well as electron and confocal microscopy [24–34]. The structure of the CNS of females and dwarf males of several species of *Osedax* was described by combining immunohistochemistry with confocal microscopy [35, 36]. In Frenulata only the general structure of mostly the central part of the nervous system is to date known based on histological and histochemical studies of the ventral nerve cords, neuropil, and brain area of a few species of the genera *Siboglinum*, *Nereilinum*, *Polybrachia,* and *Spirobrachia* [37–39].

The peripheral nervous system in annelids is usually less studied than the central nervous system. In siboglinids these data, as well as information on the muscle system of some of the most important siboglinid organs, such as papillae, are also scarce. Some data were obtained using histochemical methods such as supravital staining with methylene blue or the Zherebtsov method for acetylcholinesterase detection [39], as well as immunocytochemical data on FMRFamide and CGRPergic elements [30, 40]. They substantially contribute to our knowledge of the general anatomy of the frenulate nervous system but say almost nothing about the structure of its peripheral part and cytoarchitectonics. Electron microscopic evidence is very scarce [41, 42]. In the basal part of the epidermis, numerous neuronal projections were found, but their connections with cells were not traced. Moreover, no neuromuscular contacts or synapses were found, and no nerve elements were traced outside the basement membrane.

Ultrastructural studies revealed the presence of glial and sensory elements in the epidermis of frenulates [41] and vestimentiferans [26]. However, almost nothing is known about receptors, an important component of the peripheral nervous system. The most reliable data are only on photoreceptors of the phaosomal type, which form a local group at the anterior end of the adults and larvae of three frenulate species [43–45]. The only other sensory cells reported to date are on the opisthosoma of one frenulate species where they form two groups of sensory cells on each segment [37]. They are likely to be mechano- or chemoreceptors and could be involved in the coordination of burrowing activity. Data on other receptors are scarce and contradictory.

An almost complete lack of information on the structure of sensory systems in pogonophorans complicates comparison with other groups of annelids. The peripheral nervous system together with its sensory elements is the most evolutionarily labile component of the nervous system of animals and to a considerable degree reflects their mode of life and behavioral traits [19]. In order to provide new comparative data, we studied the adult and larval peripheral nervous system and sensory elements of the frenulate siboglinid *Oligobrachia haakonmosbiensis* Smirnov, 2000, with confocal laser scanning microscopy and immunoreactivity of five neurotransmitters. Complimentary data from scanning electron microscopy were also added. The results were compared with previous data on other siboglinids and the evolution of the nervous system within Pogonophora is discussed.

## Results

**Fig. 1:**
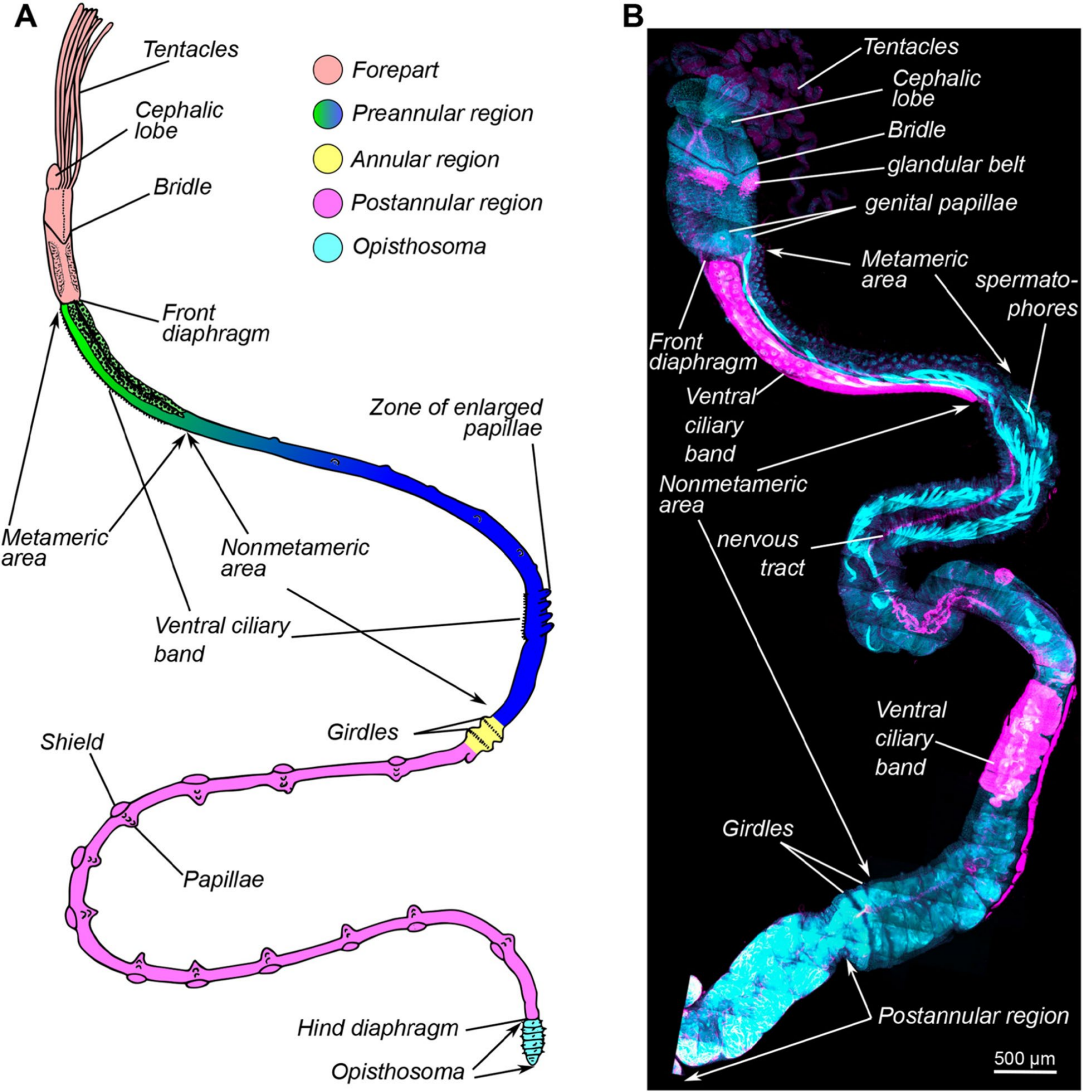
Schematic drawing (**a**) and panoramic confocal laser micrograph (**b**) of general morphology of *Oligobrachia haakonmosbiensis* highlighting main organs and regions of the body. The opisthosoma is not shown on **b.** Acetylated α-tubulin immunoreactivity (magenta) and DAPI (light blue) nuclear counterstaining

**Fig. 2:**
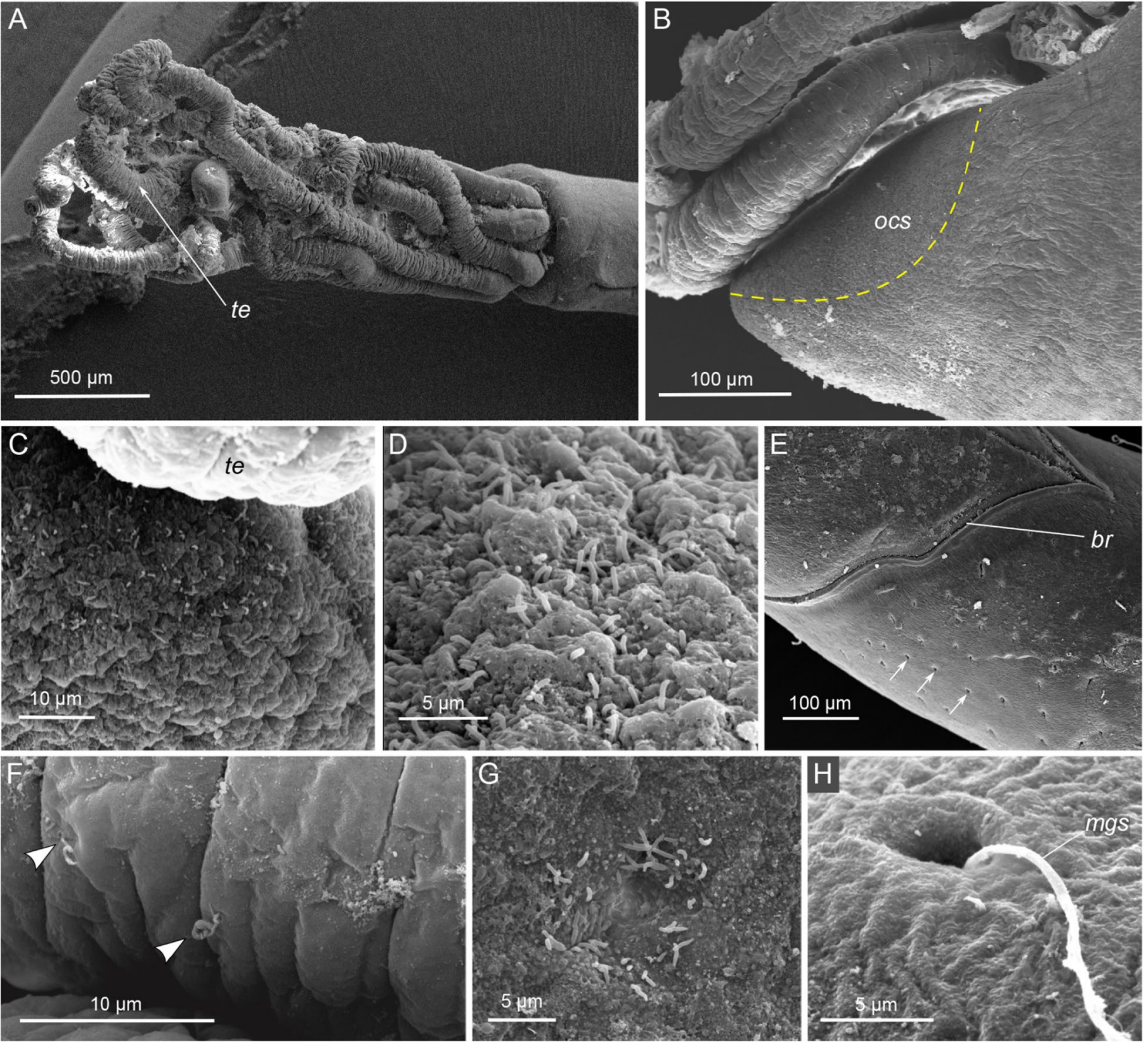
Scanning electron micrographs of the forepart and the tentacles showing ciliary structures and skin glands. **a.** The tentacular crown, dorsal view. **b.** Lateral view of the ciliated spot of epidermis (*outlined by the stippled line*) on the cephalic lobe. **c**, **d.** The same area at higher magnifications. **e.** The bridle area in dorso-lateral view showing numerous openings (*arrows*) of multicellular glands. **f.** Ciliary cells of the tentacle (*arrowheads*). **g.** Cilia around the opening of the duct of the multicellular gland at the forepart. **h.** The same showing the band of secret of the multicellular gland. Abbreviations: *br* - bridle, *mgs *– secret of the multicellular gland, *ocs* – oval ciliary spot, *te* – tentacles

As in all pogonophorans, the body of *Oligobrachia haakonmosbiensis* is subdivided into three main regions: forepart, trunk, and opisthosoma (Fig. 1). The forepart bears a cuticular ridge (the bridle) and a cephalic lobe with a crown of dorsal tentacles arising from the base of the lobe. The number of tentacles varies from 8 to 12 in different individuals (Fig. 2 a). The bridle is formed by fused cuticular plaques. The next part of the body, the trunk proper, which is subdivided into the preannular, annular, and postannular regions, is separated from the forepart by a muscular diaphragm (Fig. 1). The preannular region is marked by a ventral ciliary band that begins at its anterior end. The dorsal side of the body above the ciliary band bears papillae, specialized extensions of the body wall tipped with cuticular plaques. This part of the preannular region, which is called the metameric area, is followed by the nonmetameric area of the preannular region. The dorsal side of the nonmetameric area carries irregularly arranged solitary papillae. A small group of enlarged papillae is located near the posterior end of this area. Another, small patch of the ciliary band is present below this group of papillae, on the ventral side of the body.

The postannular region of the trunk is characterized by the presence of regularly arranged dorsal rows of papillae and the glandular shields positioned opposite to the papillae on the ventral side. The shields are formed by a thickened epithelium and lack cuticular plaques. The body ends with the opisthosoma, which is composed of several small body segments, however the studied individuals lost their opisthosoma regions during the collection of the material.

### Scanning electron microscopy of adult *O. haakonmosbiensis*

On the dorsal side of the cephalic lobe in front of the tentacles the oval spot is located, which bears a great number of solitary short cilia of uniform morphology (Fig. 2 b–d). Here the cilia are evenly distributed between the apical surfaces of epithelial cells. As the cilia arise from depressions between the epithelial cells, their full length cannot be measured. Their length above the epithelial surface is, on average, 2 μm. All tentacles also bear evenly distributed solitary cilia that form rows along the length of the tentacles (Fig. 2 f). These cilia are much longer, about 5 μm, and usually appear coiled.

**Fig. 3:**
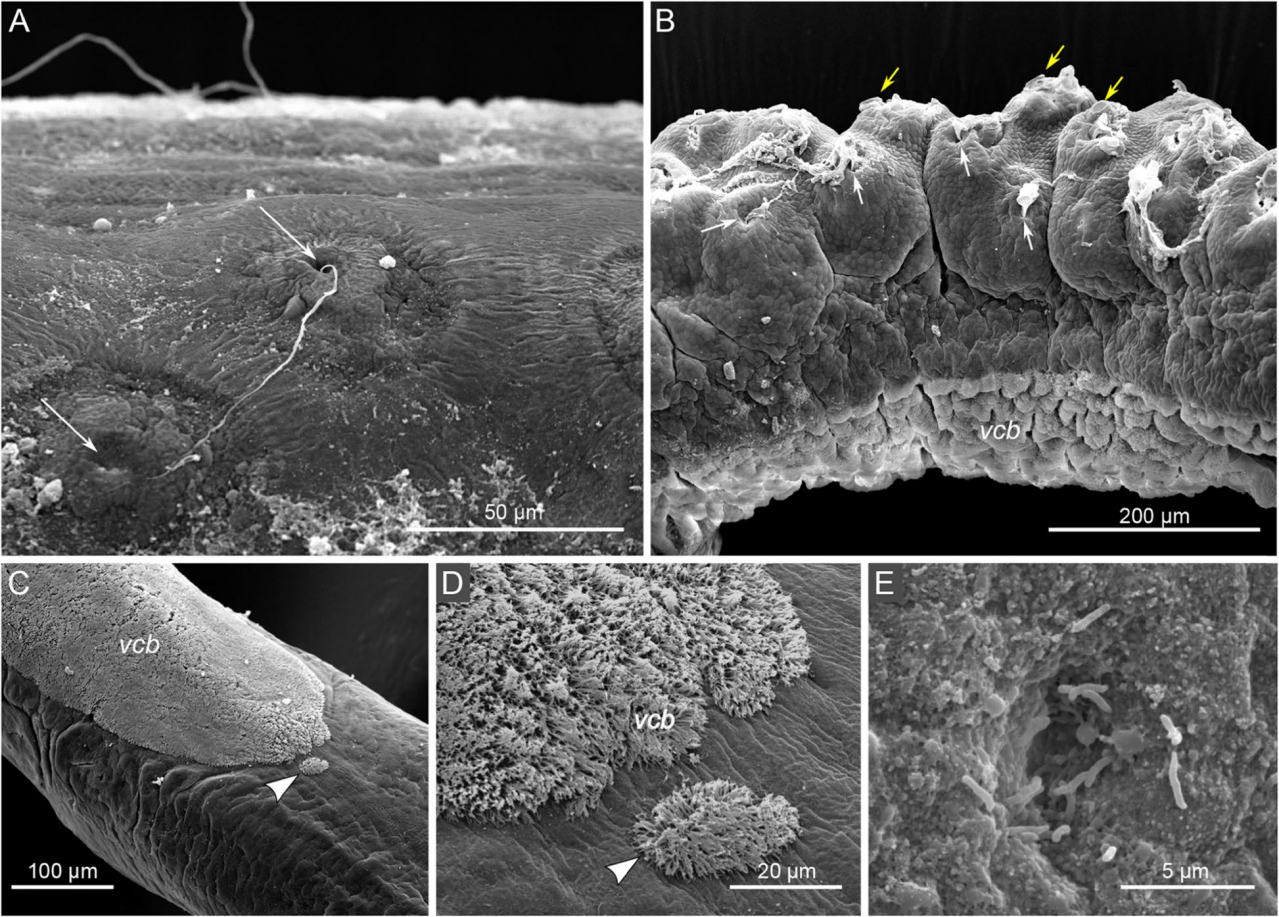
Scanning electron micrographs of the metameric area of the trunk. **a.** The anterior part of the metameric area showing dorso-lateral epidermal-muscular ridges with openings of multicellular glands (*arrows*). **b.** The posterior part of the metameric region showing ventral ciliary band, faintly separated dorsal papillae with cuticular plaques (*yellow arrows*), and openings of multicellular glands (*white arrows*). **c.** The posterior part of the ventral ciliary band with a small detached area (*arrowhead*). **d.** The same area at higher magnification. **e.** Cilia around the opening of the duct of the multicellular gland on the metameric papilla. Abbreviations: *vcb* – ventral ciliary band

**Fig. 4:**
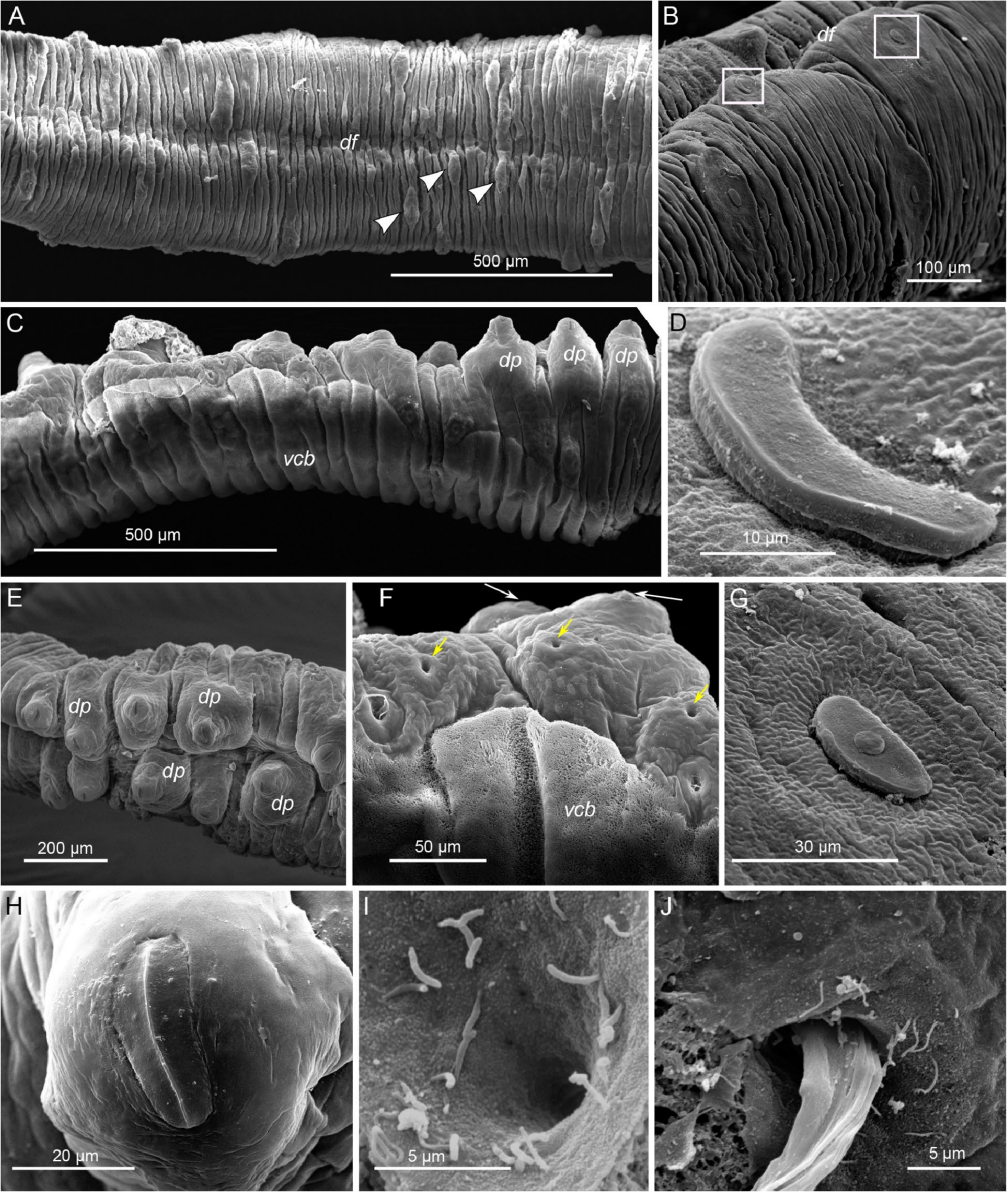
Scanning electron micrographs of the nonmetameric area of the trunk. **a.** Panoramic view of the nonmetameric area showing small scattered dorsal papillae (*arrowheads*). **b.** The same area at higher magnification showing cuticular plaques (*squares*). **c.** The zone of enlarged dorsal papillae and ventral ciliary band. **d, g.** The cuticular plaques of the nonmetameric papillae. **e** Enlarged papillae in dorsal view. **f.** The same area at higher magnification showing cuticular plaques (*white arrows*), openings of multicellular glands (*yellow arrows*) and ventral ciliary band. **h.** The tip of the enlarged papilla with the cuticular plaque. **i.** Cilia around the opening of the duct of the multicellular gland on the enlarged papilla. **j.** The same on the nonmetameric papilla. Abbreviations: *df *– dorsal furrow, *dp* – dorsal papilla, *vcb* – ventral ciliary band

The dorsal surface of the forepart below the tentacular bases forms a furrow in front and behind the bridle (Fig. 1). Below the bridle, numerous ducts of multicellular tubiparous glands, which are common in the pogonophorans, open dorsolaterally on each side of the dorsal furrow posterior to the diaphragm (Fig. 2 e). These glands are known to be composed of a long duct passing through the muscle layers and epidermis and a secretory portion that bulges out into the coelom [37]. Their secretion is released as long ribbons (Fig. 2 h; Fig. 3 a; Fig. 4 j). Relatively short, solitary cilia surround the opening of each gland (Fig. 2 g, h). The bases of cilia are lined with a thin layer of mucus, which cannot be removed entirely without damaging the epithelial surface and cilia. The length of the cilia above the mucus is, on average, 2–3 μm.

As mentioned above, the metameric area of the preannular region bears numerous papillae on its dorsal surface. At the anterior end of the metameric area, they are lower and are almost flush with the body surface forming dorsal epidermal-muscular ridges (Fig. 3 a). Further posteriorly, the papillae become more pronounced and appear as two ridges on either side of the dorsal furrow that continues posteriorly beyond the diaphragm (Fig. 3 b). Cuticular plaques on the papillae in this area are round with a thickened anterior edge that projects over the surface of the papilla and forms a ridge. On the tip of each papilla near the cuticular plaque the duct opening of a tubiparous gland is located. As on the forepart, each opening is surrounded by 12–20 solitary cilia (Fig. 3 e).

An interesting observation was made while examining the ventral ciliary band of the metameric area: a small cluster of ciliary cells was found at some distance from the main band to form a small additional portion of the band (Fig. 3 c, d).

In the nonmetameric area, the body forms numerous circular folds, whose apices on the dorsal side bear small rare solitary scattered papillae (Fig. 4 a, b). Cuticular plaques of these papillae are often round or sometimes kidney-shaped (Fig. 4 d, g). As in the metameric area, the ducts of the tubiparous glands open near the cuticular plaques. The so-called enlarged papillae project from the body surface above the second portion of the ventral ciliary band in the preannular body region (Fig. 4 c, e, f). The cuticular plaques on their apices are oval and bear a thickened ridge along their midline (Fig. 4 h). As in the other regions of the body, duct openings of tubiparous glands are present on each papilla, but here they are located closer to their bases. The openings are surrounded by several solitary cilia. The shape, length, and number of these cilia are generally consistent with those found on other papillae of the metameric area (Fig. 4 i, j).

**Fig. 5:**
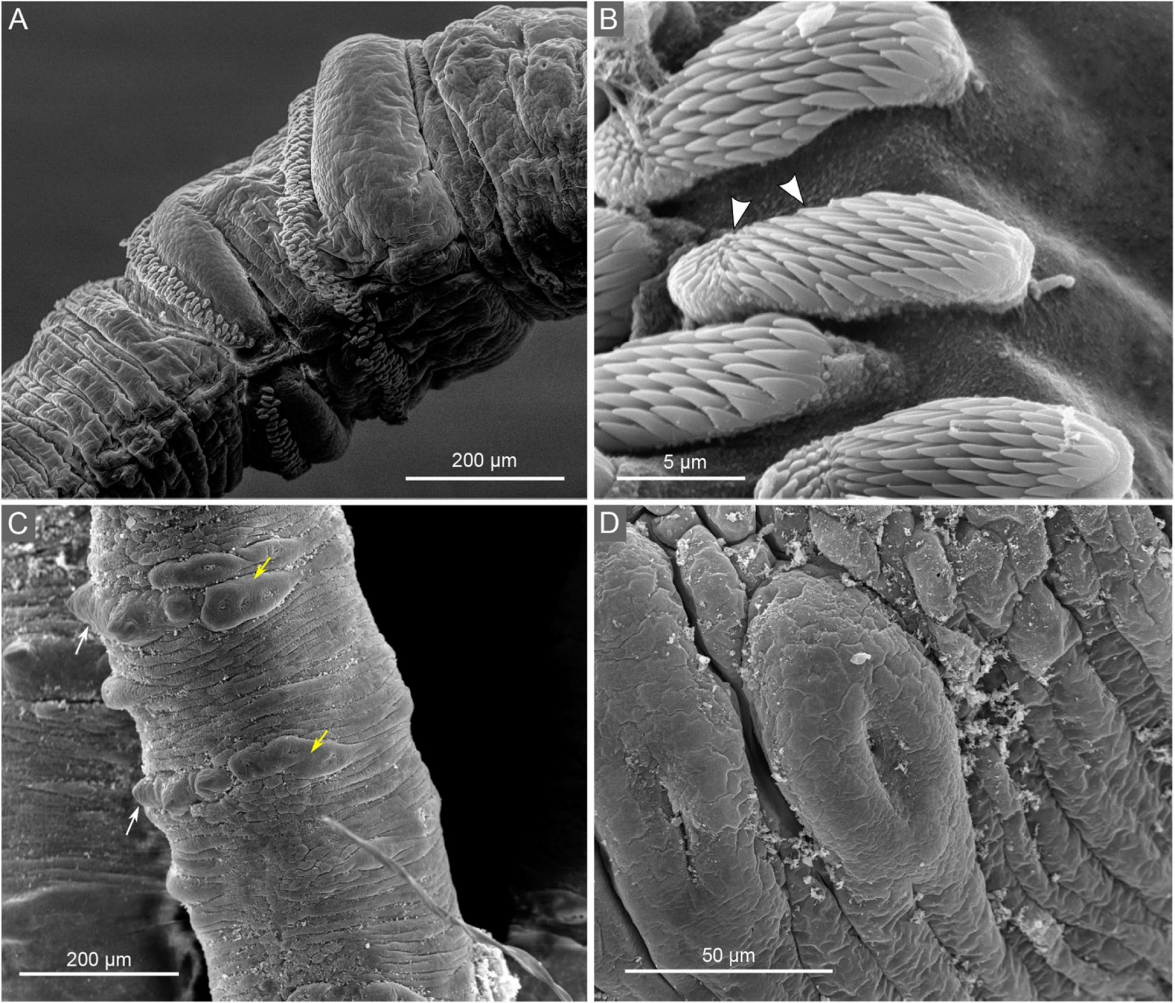
Scanning electron micrographs of the annular and the postannular regions of the trunk. **a.** Panoramic view of the annular region showing two girdles of toothed chaetae. **b.** The heads of the toothed chaetae showing two opposite groups of denticles (*arrowheads*). **c.** Panoramic view of the postannular region showing ventral glandular shields (*yellow arrows*) and dorsal ridges of papillae (*white arrows*). **d.** The ventral glandular shields with openings of ducts of multicellular glands

The preannular region is followed by a short annular zone bearing two circular muscular girdles with chaetae; this zone marks the boundary between the preannular and postannular regions (Fig. 1; Fig. 5 a, b). Each chaeta ends in a flattened head that protrudes above the body surface and carries two opposite groups of teeth, a smaller group located anteriorly and a larger one posteriorly (Fig. 5 b). Papillae of the postannular region are arranged in transverse rows on the dorsal body side. The apices of the papillae bear cuticular plaques similar to those on the papillae of the nonmetameric area. The duct of a tubiparous gland, also encircled by cilia, opens near the plaque on each papilla. On the ventral side of the body opposite the papillae lie the glandular shields associated with tubiparous glands but lacking the cuticular plaques (Fig. 5 c, d).

### Scanning electron microscopy of larvae of *O.haakonmosbiensis*

**Fig. 6:**
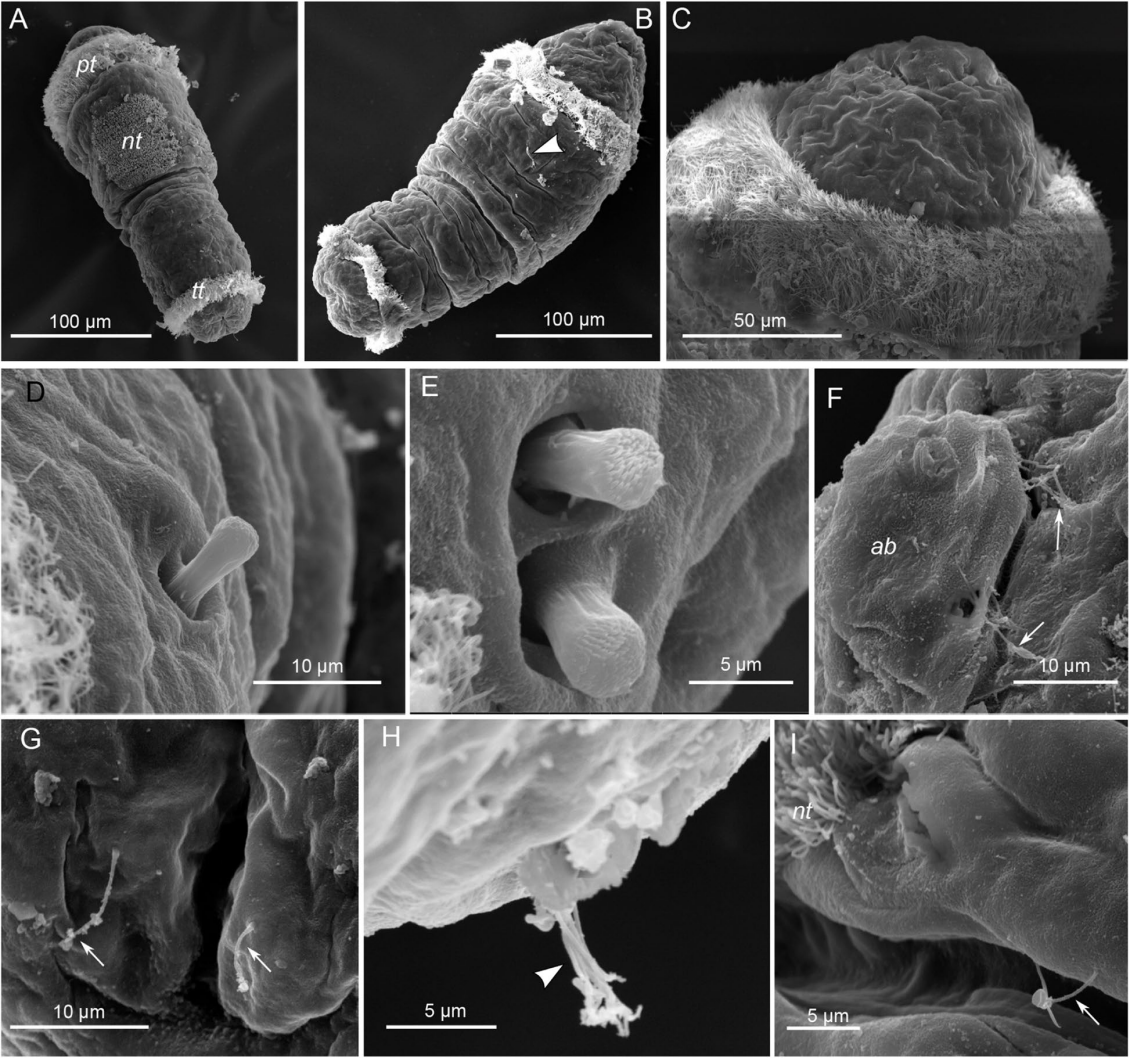
Scanning electron micrographs of larvae of *Oligobrachia haakonmosbiensis*. **a.** Panoramic view of a larva showing two ciliary rings, the anterior prototroch (*pt*) and the posterior telotroch (*tt*), and the ventral ciliary band, neurotroch (*nt*). **b.** The same from the dorsal side. **c.** The extremely anterior tip of a larva showing the prototroch and no apical tuft. **d.** One chaeta on the larval opisthosoma. **e.** Two chaetae on the larval opisthosoma. **f.** Cilia (*arrows*) around the apical bulge. **g.** High-magnification inset of the bottom of (**a**) showing the bilobed pigidium with one cilium on each lobe (*arrows*). **h.** Clumped cilia (*arrowhead*) on the dorsal side of the larva behind the prototroch. **i.** Solitary cilia scattered through the body of the larva (*arrow*). Abbreviations: *ab* – apical bulge, *nt* – neurotroch, *pt *– prototroch, *tt* – telotroch

All larvae removed from the tubes were at the trochophore stage and only slightly differed in age, which was evident in a different length of the neurotroch and a different degree of chaetal development (Fig. 6). The chaetae probably do not develop simultaneously. In younger larvae, in which the neurotroch covers a small area, only one chaeta is present on either side (Fig. 6 d), while the larvae with a larger neurotroch have two chaetae on each side of the body (Fig. 6 e).

The prototroch and telotroch are well developed in all larvae (Fig. 6 a–c). The apical ciliary tuft is lacking (Fig. 6 c), but a few isolated cilia (~ 10 μm in length) can be seen at the base of the apical bulge (Fig. 6 f). Two solitary cilia are located symmetrically on the pygidium, one on each side of the median furrow (Fig. 6 g). The length of these cilia is 6–7 μm. A bundle of 5–7 clumped cilia arises from a small epithelial pit on the dorsal side of the larva behind the prototroch (Fig. 6 b, h). The length of the cilia in this structure measured above the epithelial surface is 8 μm. A few solitary cilia, about 5 μm in length, are scattered between the proto- and telotroch (Fig. 6 i).

### Immunohistochemistry and histochemistry

Four of the antibodies used in this study (against acetylated α-tubulin, substance P (SP), serotonin (5-hydroxytryptamine or 5-HT), and FMRFamide) showed specific reactivity to different parts of the nervous system. At the same time, anti-octopamine antibody revealed probably only a part of specific nerve elements, because of improper fixation (4% paraformaldehyde solution (PFA) instead of PFA with 0.3% glutaraldehyde recommended by the antibody manufacturer).

Acetylated α-tubulin immunostaining showed that in *O. haakonmosbiensis* this antibody can visualize not only ciliary structures and neurites of the nerve cells, but also numerous neuronal somata and, to a certain degree, receptor cells in the peripheral nervous system. All somata of neurons and sensory cells as well as most of the visualized branches of their neurites lie within the epidermis. The exceptions are those neurites that participate in the innervation of the internal structures of the body and musculature; these neurites extend below the epithelium into the interior of the body.

#### Cephalic tentacles

**Fig. 7:**
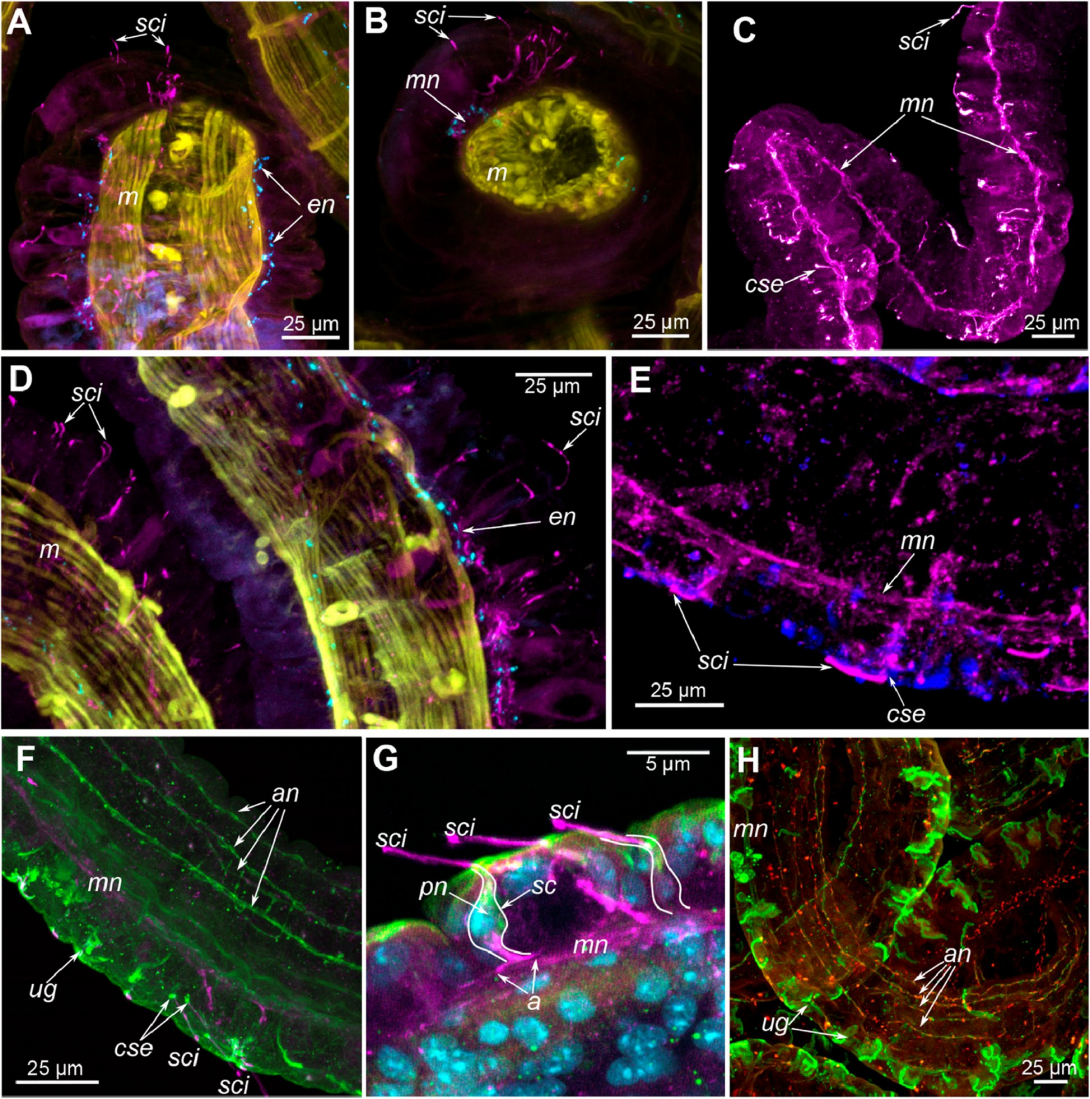
Sensory and neuronal elements of the tentacles. **a.** Longitudinal view on tentacle showing tentacular musculature (yellow) supported by FMRFamide-lir fibers (cyan). **b.** An optical cross-section through the same tentacle. **c.** Sensory elements of the main tentacular nerve revealed by acetylated α-tubulin-lir. **d.** Sensory elements and FMRF-like innervation in tentacular epithelium and musculature. **e.** Monociliated sensory ending of octopamine-lir cells. **f.** SP-lir sensory elements and nerves of tentacles. **g.** SP-lir sensory cells of the tentacular epithelium. The cell somata are outlined. **h.** 5-HT-lir fibers of tentacular nerves. Abbreviations: *a* – axon, *an* – additional tentacular nerves, *cse* – monociliated sensory ending, *en* – efferent neurites, *m* – tentacular muscles, *mn* – main tentacular nerve, *pn* – perikaryon, *sc* – sensory cell, *sci* – sensory cilia, *ug* – unicellular glands

All cephalic tentacles are similar in morphology. They are lined with a high cylindrical epithelium underlain by outer circular and inner longitudinal muscle layers. Each tentacle contains a central coelomic channel. The epithelium of the tentacles is abundantly supplied by unicellular glands. Most of these glands are arranged in a weakly pigmented longitudinal band that extends from the base to the tip of each tentacle. Glandular cells lie separately and are interspersed by epithelial and sensory cells (Fig. 7). Each tentacle has 5 nerves (Fig. 7 f, h). Four of them can be traced to the extreme tip of the tentacle, where they thin down and form a common nerve plexus. The neurites of these nerves stain positively for acetylated α-tubulin, SP, FMRFamide, and 5-HT. At a cross-section, all nerves are shifted somewhat toward one half of the tentacle. The nerves form anastomoses because some neurites or their collaterals pass into the adjacent nerves (Fig. 7 f, h; Additional Fig. 1 a, b). The largest (main) nerve is situated at the base of the epithelium of the pigmented band (Fig. 7 c, e, f). This nerve is formed primarily by axons of numerous monociliary sensory cells that are located in the vicinity of the nerve. Their cilia are about 20 μm in length. The perikarya and some of the processes of these cells stain positively for both acetylated α-tubulin and SP, while others only for one of those (Fig. 7 a–d, f–h; Additional Fig. 1 b). A small number of monociliary cells are labeled by octopamine antibody (Fig. 7 e). In sensory cells, anti-tubulin antibody staining often reveals only a portion of the perikarya with a short dendrite carrying a single cilium (Fig. 7 a–d, g). Similar solitary sensory cells are present in the epithelium and near other nerves of the tentacles. The axons of the sensory cells join these nerves and project toward the CNS.

Muscles – yellow (phalloidin staining), acetylated α-tubulin-lir – magenta, FMRFamide-lir – cyan (a, b, d), Octopamine-lir – blue (e), 5HT-lir – red, Substance P-lir – green, cell nuclei – light blue (DAPI staining) (g).

FMRFamide was found in only a few neurites in two small tentacular nerves (Fig. 7 a, d) and is sometimes also present in a few neurites in the large nerve (Fig. 7, b). These neurites leave the epithelium to form terminal arborizations in the musculature of the tentacles. Varicosities and terminals that contain FMRFamide-related peptide are clearly seen in association with muscle fibers (Fig. 7 a, b, d). 5-HT-like immunoreactivity was revealed in only a few neurites within the four tentacular nerves (Fig. 7 h). A series of bead-like varicosities are arranged along the entire length of these neurites. Terminal arborizations of 5-HT-immunoreactive neurites were observed as free nerve endings at the unicellular glands and among the epithelial cells. No perikarya associated with FMRFamide or 5-HT neurites were found in the tentacles. They are probably located in the CNS.

#### Forepart

**Fig. 8:**
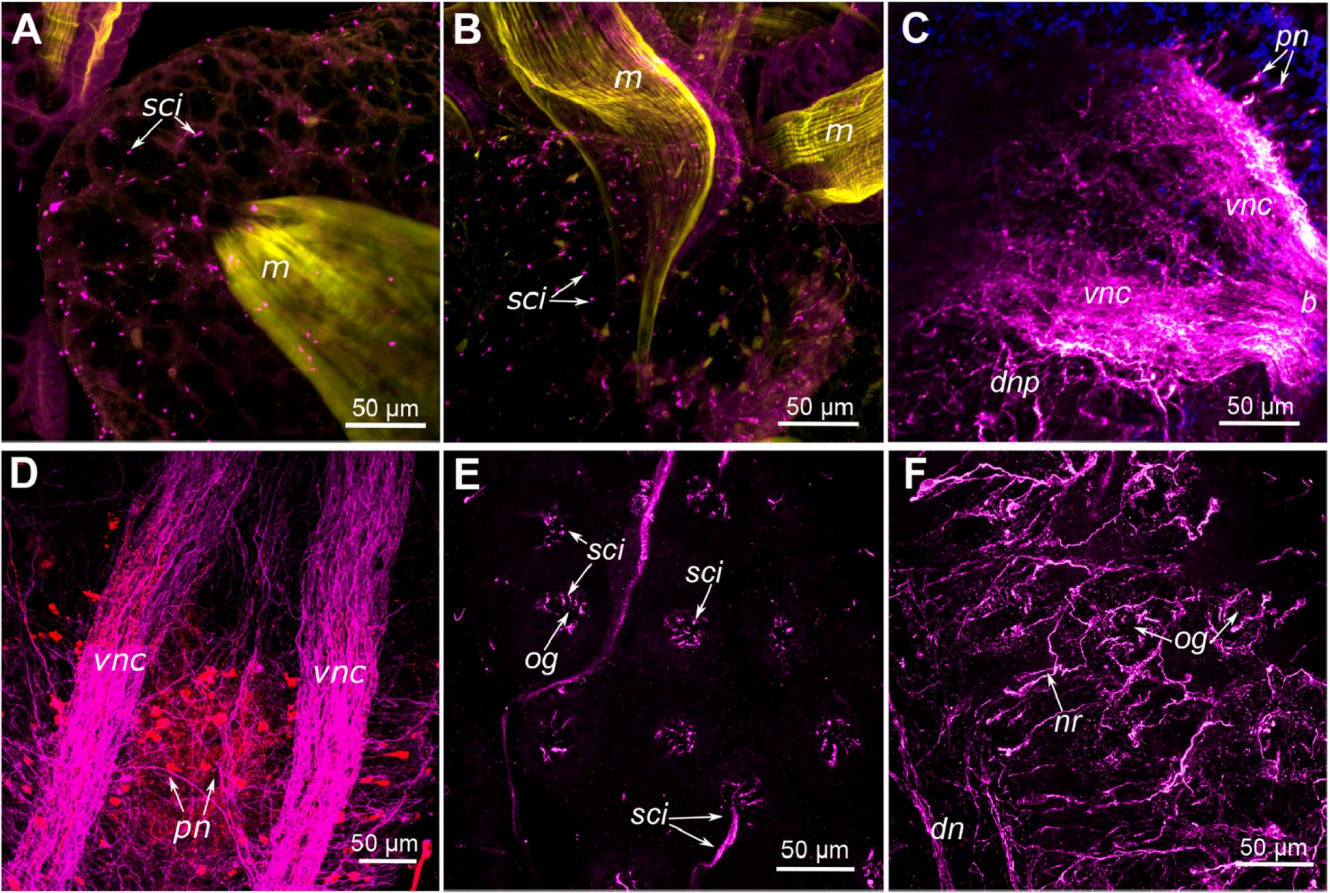
Sensory and neuronal elements of the forepart. **a, b.** Sensory area of the cephalic lobe at the base of tentacles. **c.** Forepart before the bridle, ventral view. Nerve plexus of the body wall adjacent to the ventral nerve cords. **d.** Forepart after the bridle ventral view. **e, f.** Successive partial Z-projections of the body wall at the area of the dorsal furrow showing openings of tubiparous glands surrounded by sensory cilia (**e**), and innervation of tubiparous glands by dorsal nerves (**f**). Abbreviations: *b* – bridle, *dn* – dorsal nerve, *dnp* – diffuse nerve plexus, *m* – musculature, *nr* – neurites, *og* – tubiparous gland duct opening, *pn* – perikaryon, *sci* – sensory cilia, *vnc* – ventral nerve cord. Muscles – yellow (phalloidin staining), acetylated α-tubulin-lir – magenta, 5HT-lir – red, cell nuclei – blue (DAPI staining)

The acetylated α-tubulin antibody revealed the area containing a significant number of evenly distributed solitary short cilia on the dorsal side of the cephalic lobe near the tentacular bases (Fig. 8 a, b). These observations agree with the results obtained by scanning electron microscopy (Fig. 2 b–d). None of the applied antibodies labeled somata of the cells bearing these cilia.

**Fig. 9:**
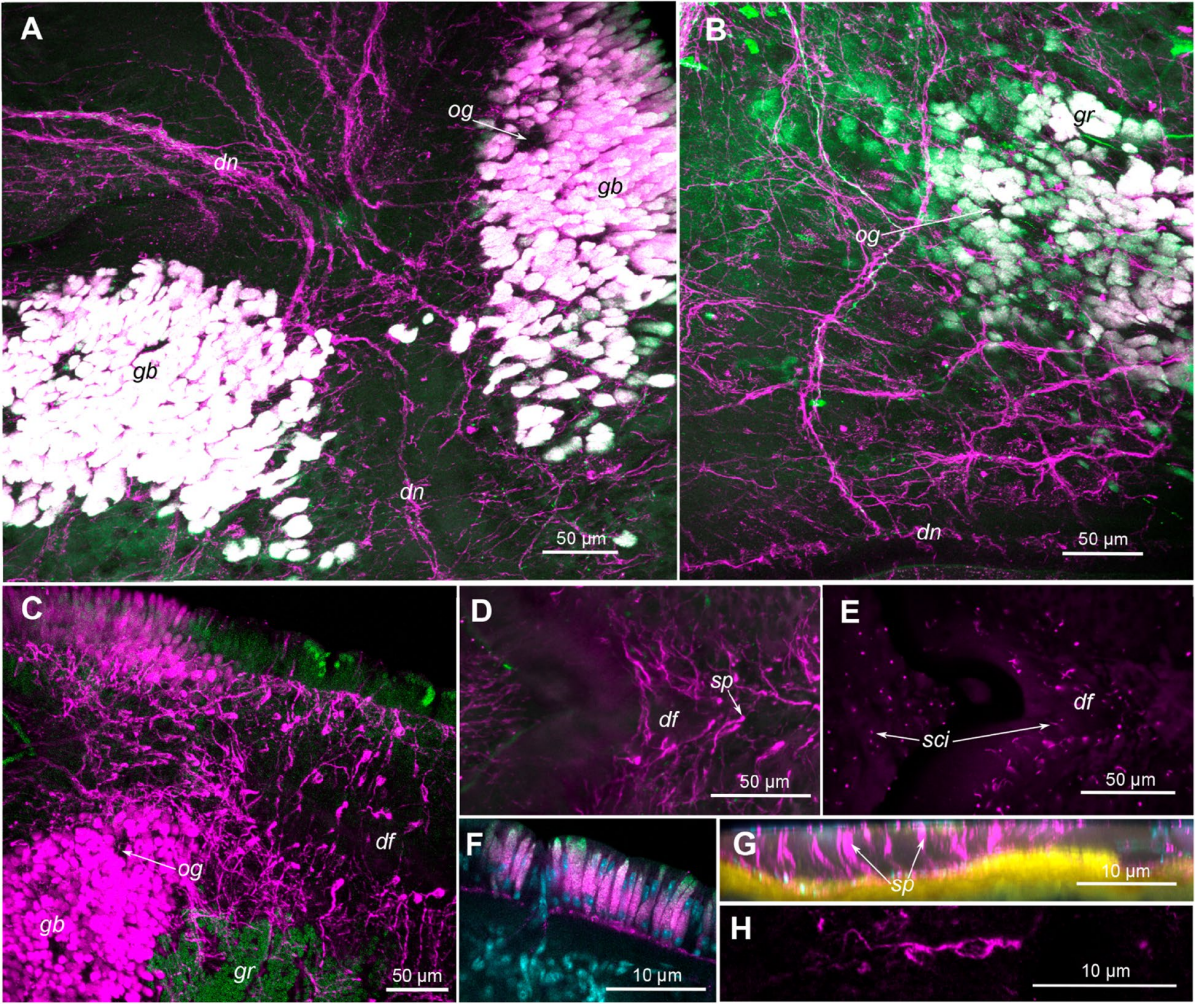
Forepart behind the bridle and the innervation of the dorsal furrow. **a, b, c.** Innervation of the two types of glands (glandular belt and glandular ribbon) in the forepart by the dorsal nerve. **d, e, g.** Sensory cells of the dorsal furrow. Successive partial Z-projections showing the plexus (**d**) and sensory cilia (**e**). **g.** A virtual sagittal section through the same region. **f.** Secretory cells of the glandular belt with underlying neurites, virtual sagittal section. **h.** A neuron from the area of the dorsal furrow. Abbreviations: *df* – dorsal furrow, *dn* – dorsal nerve, *gb* – glandular belt, *gr* – glandular ribbon, *og* – opening of the gland duct, *sci* – sensory cilia, *sp* – sensory cell perikarya. Acetylated α-tubulin-lir – magenta, 5HT-lir – green, muscles – yellow (phalloidin staining), cell nuclei – light blue (DAPI staining)

An extensive diffuse intraepithelial nerve plexus was visualized on all sides of the forepart using anti-acetylated α-tubulin antibody. Many of the neurites composing this plexus are associated with the ventral nerve cords (Fig. 8 c–d; Additional Fig. 1 c). The neurites of the ventral and lateral nerve plexuses do not form any distinct nerves. Most of them enter the ventral nerve cords separately. The latter converge in the bridle area and diverge again posteriorly (Fig. 8 c, d). Many of the neurites of the ventral nerve plexus belong to the unipolar neurons distributed diffusely in the same plexus (Fig. 8 с, d). The nerve plexus on the dorsal side is most developed in the area of the dorsal furrow (Fig. 8 f; Fig. 9 c). Above the diaphragm this plexus consists of several thin loosely structured nerves that converge together as they pass through the area of the bridle (Fig. 9 a). The dorsal nerve plexus is most extensive below the bridle and participates in the innervation of specialized glandular structures located symmetrically on both sides of the dorsal furrow (Fig. 9 a–c). Below the dorsal furrow, the plexus consists of several small longitudinal nerves (Fig. 8 f; Fig. 9 a, b). Two pairs of glandular structures composed of large tight clusters of numerous intraepithelial secretory cells lay immediately below the bridle area and spread to some extent to the lateral sides of the forepart. The secretory cells are interspersed with thin layers of epithelial cells and the ducts of subepithelial multicellular tubiparous glands (Fig. 9 a–c, f). The arrangement and appearance of the duct openings of these subepithelial glands were described by scanning electron microscopy (Fig. 2 e, h). Immediately below the bridle is a glandular structure in the shape of a split belt, whose secretory granules can be visualized by autofluorescence under the standard settings for the Alexa Fluor 633 fluorochrome. Another paired glandular structure in the form of longitudinal ribbons is located immediately behind the first, closer to the diaphragm. The cells of this structure in the same specimens were visualized under the standard settings for the Alexa Fluor 488 fluorochrome (Fig. 9 c).

Monociliary receptor endings surround the duct openings of the tubiparous glands (Fig. 8 e), as also seen by scanning electron microscopy (Fig. 2 g). The nerve plexus found in the area of the dorsal furrow contains a significant number of neuronal perikarya (Fig. 9 h). It innervates the tubiparous glands and the two paired glandular structures described above (Fig. 9 c). Thе plexus also contains a large cluster of bipolar receptor cells located along the furrow between the glandular structures (Fig. 9 d, e, g). The apical ends of the dendrites arising from these sensory cells bear single cilia 8–10 μm in length (Fig. 9 е). Scanning electron microscopy did not reveal any cilia in the dorsal furrow area, but this might be explained by the presence of the mucus lining over the surface of the furrow.

The basal parts of secretory cells in the paired upper glandular structure that lies adjacent to the bridle are especially profusely innervated (Fig. 9 a, c, f). Most of these cells do not reach the epithelial surface and possess secretory granules in the basal parts. Thus, it remains unclear where they are discharged. It is still unclear whether the secretory cells themselves form the intraepithelial plexus or it is a bundle of neuronal processes, coming from some other cells. In the latter case, these glands can be neurosecretory and the presence of numerous receptor cells in the vicinity of these glands along the dorsal furrow is not accidental.

#### Nerve plexuses of the trunk

**Fig. 10:**
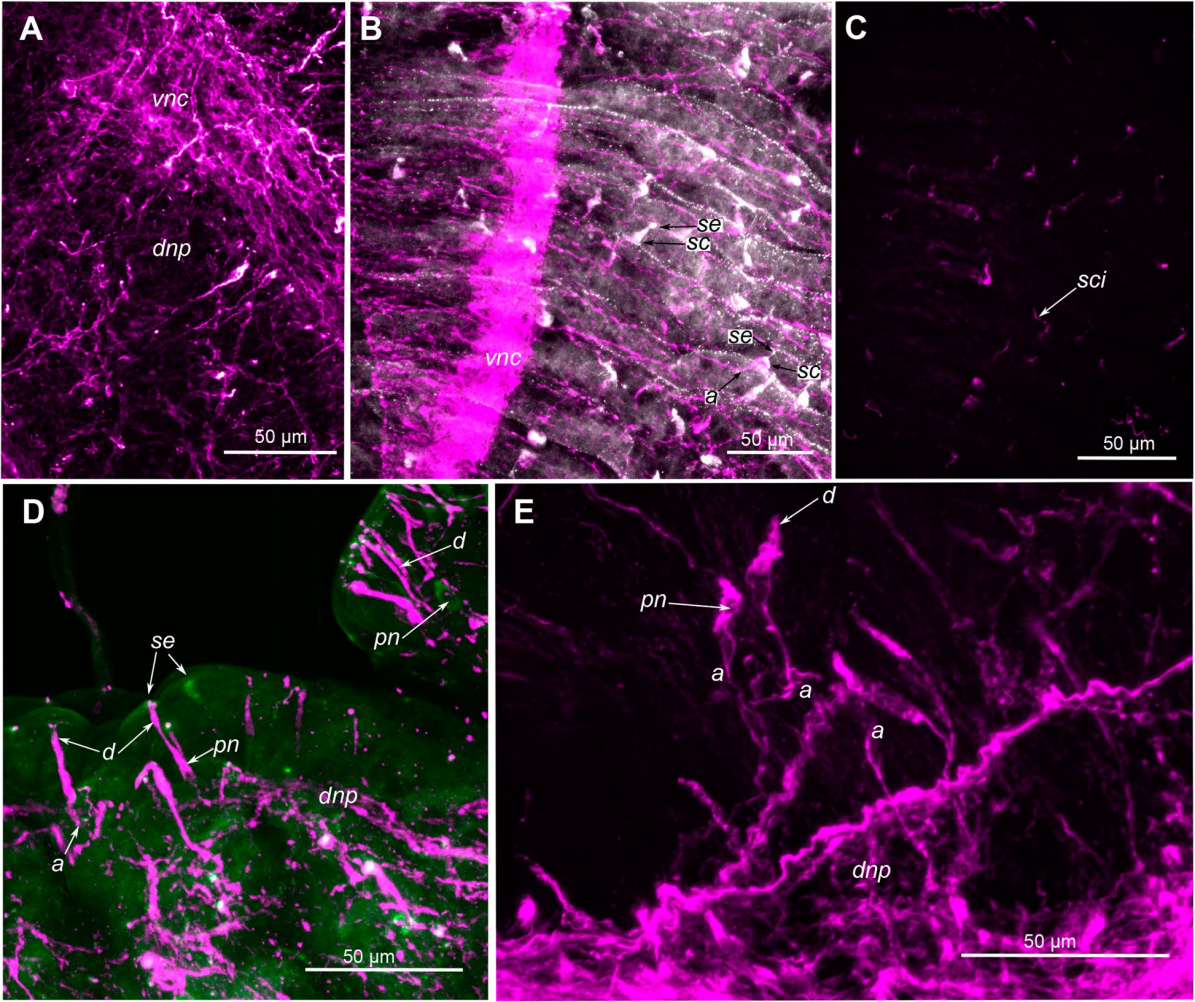
Nerve plexuses of preannular region. **a.** Diffuse nerve plexus of the body wall adjacent to the ventral nerve cord. **b.** Octopamine-lir sensory cells of the body wall. **c.** Partial Z-projection of the same confocal stack, showing sensory cilia of the cells on **b**. **d, e.** SP-lir and acetylated α-tubulin-lir sensory cells and their axons in the nerve plexus of body wall epithelium. Abbreviations: *a* – axon, *d* – dendrite, *dnp* – diffuse nerve plexus, *pn* – perikaryon, *sc* – sensory cell, *se* – sensory ending*, sci* – sensory cilia, *vnc* – ventral nerve cord. Acetylated α-tubulin-lir – magenta, Octopamine-lir – white, Substance P-lir – green

**Fig. 11:**
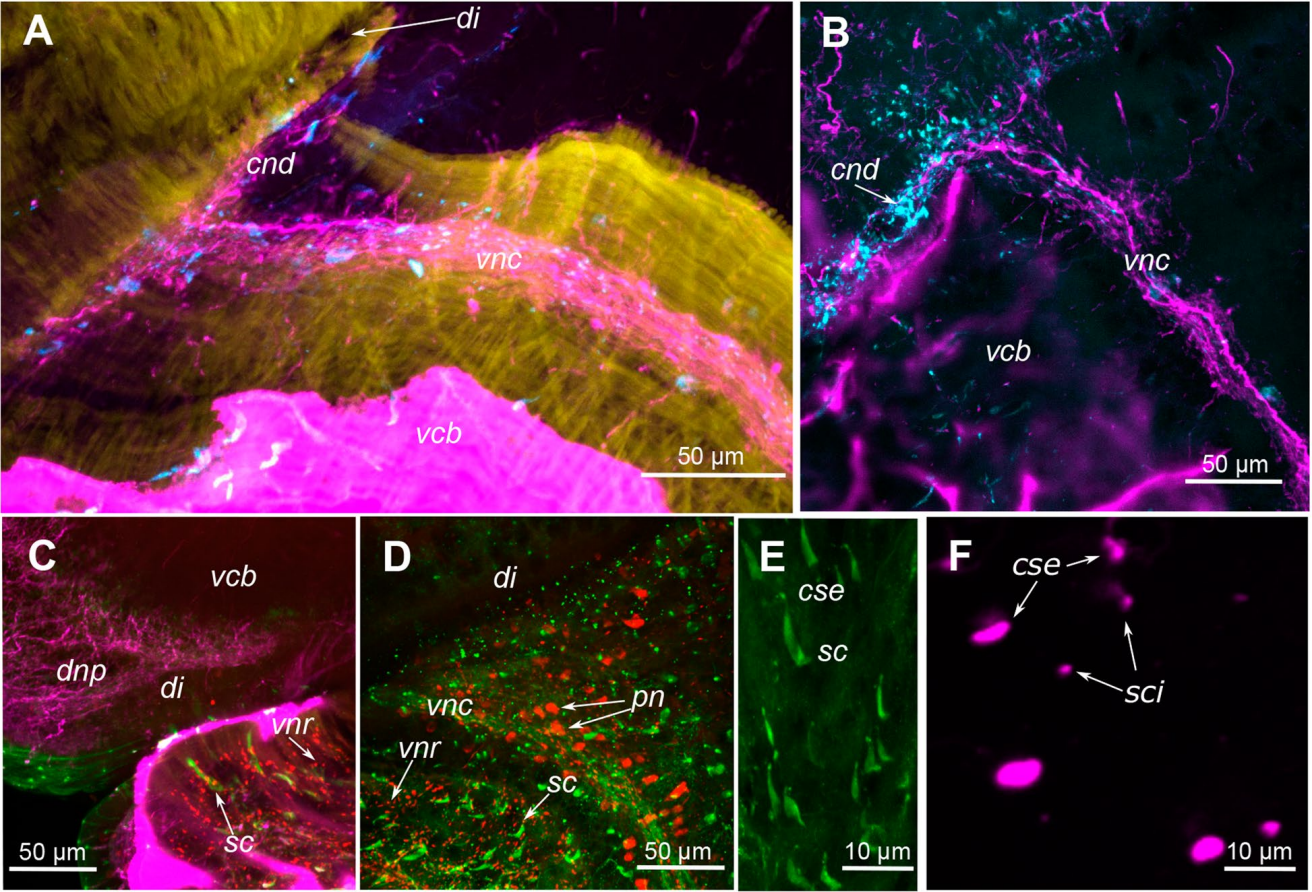
Nerve plexus of the diaphragm and ventral ciliary band. **a, b.** FMRF-lir in the commissural nerve of the diaphragm and the innervation of adjacent musculature. **c, d, e.** Different sensory cells of the ciliary band after the diaphragm showing immunoreactivity against 5HT (**c, d**) and SP (**c, d, e**). **f**. cilia of SP-lir cells from **e**. Abbreviations: *cnd* - commissural nerve of the diaphragm, *cse* – monociliated sensory ending, *di* – diaphragm, *dnp* – diffuse nerve plexus, *pn* – perikaryon, *sc* – sensory cell, *sci* – sensory cilia, *vcb* – ventral ciliar band, *vnc* – ventral nerve cord, *vnr* – varicoses of neurites,. Muscles – yellow (phalloidin staining), acetylated α-tubulin-lir – magenta, FMRFamide-lir – cyan, 5HT-lir – red, Substance P-lir – green

**Fig. 12:**
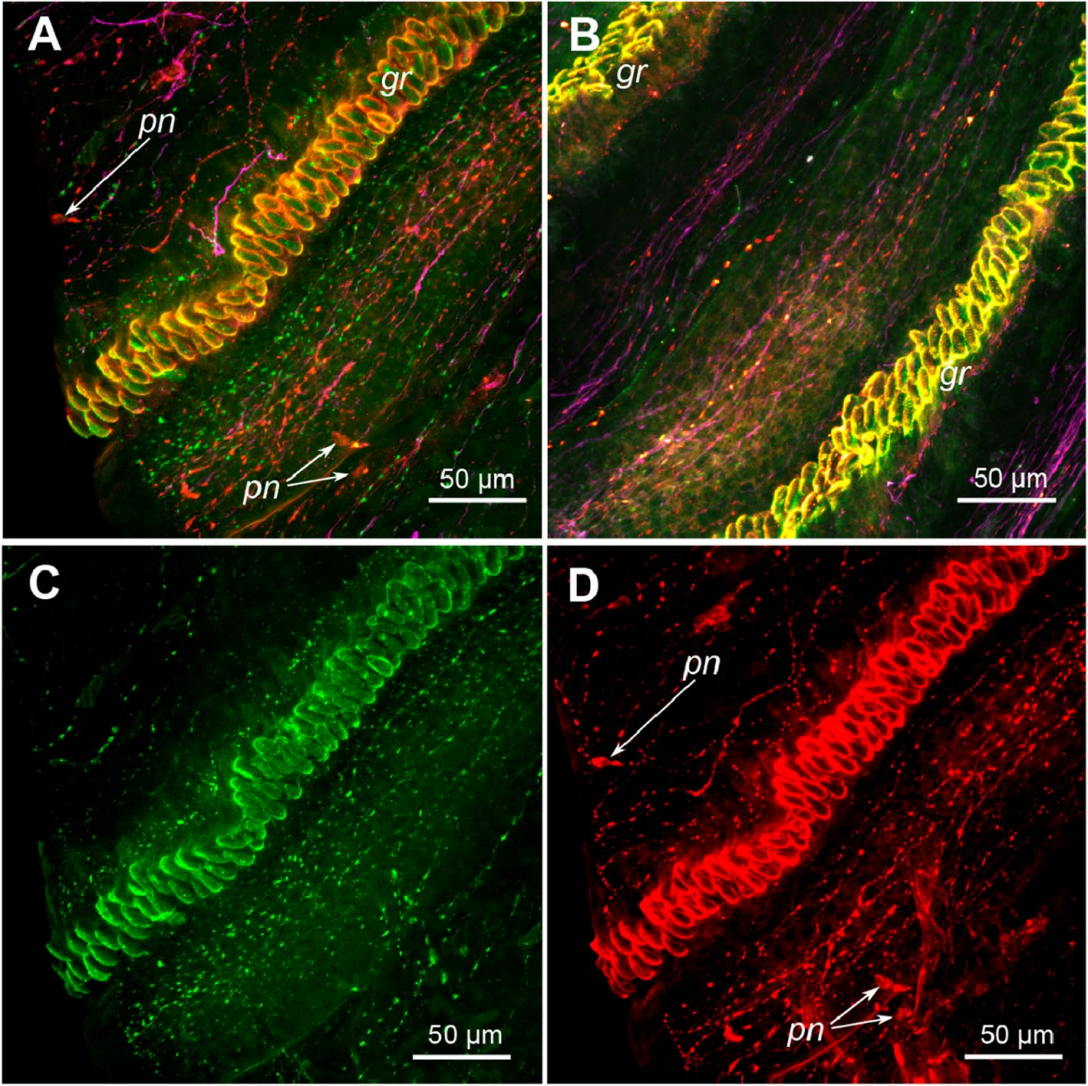
Sensory elements and innervation of girdles. **a.** Innervation of the anterior girdle, **b.** Innervation of the body wall between girdles, **c, d.** Separated fluorescence channels from **a,** showing SP-lir (**c**) and 5HT-lir elements (**d**) of the girdle region. Abbreviations: *pn* –perikarya, *gr* – girdles. Acetylated α-tubulin-lir – magenta, 5HT-lir – red, Substance P-lir – green

**Fig. 13:**
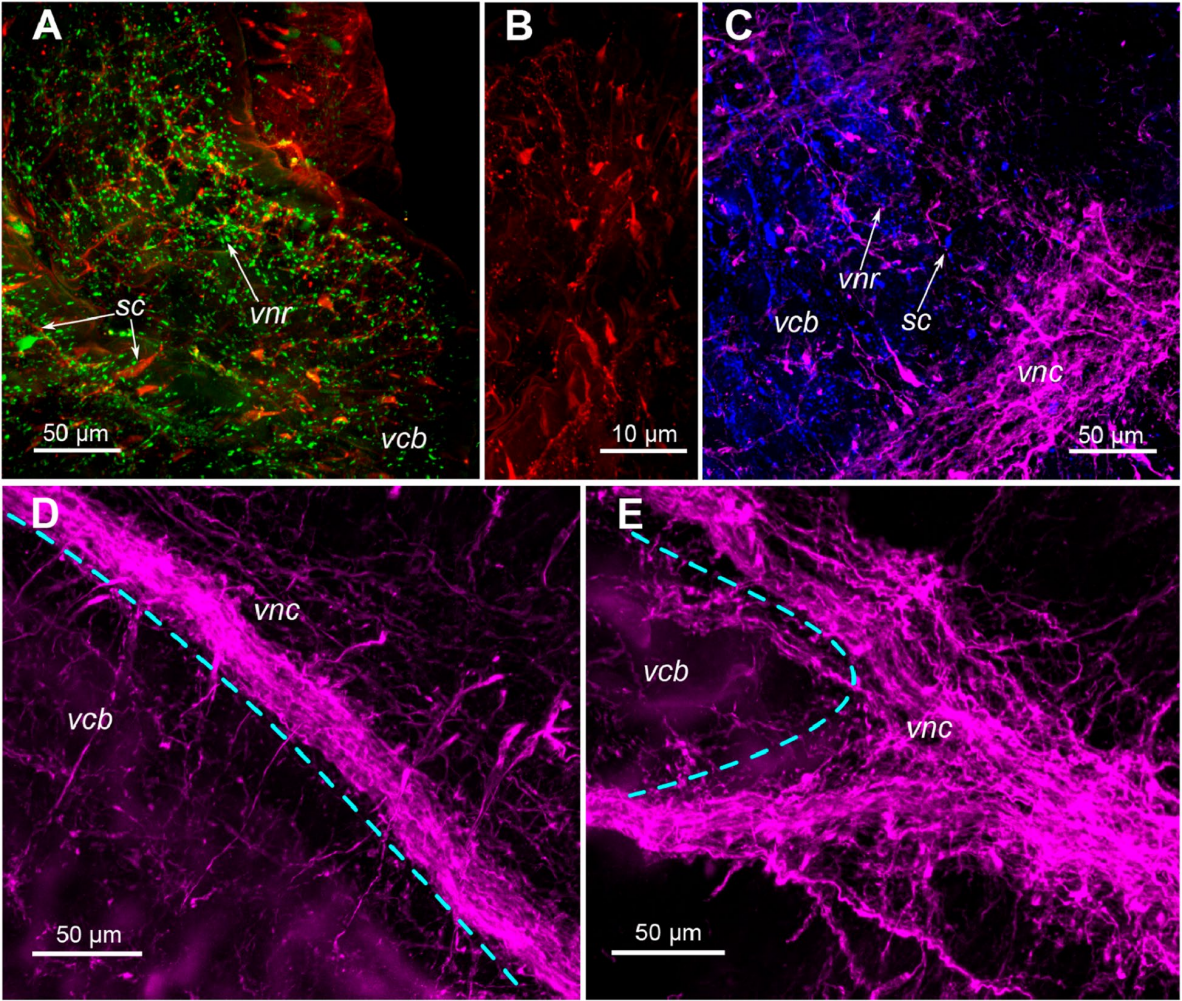
Innervation of the body wall at the area of the ciliary band. Partial z-projections, the external ciliation is not shown, see Additional Fig. 2 for more information. **a** 5HT-lir (red) and SP-lir (green) elements underlying the ciliary band**. b.** 5HT-lir sensory cells of the ciliary band, **c.** Octopamine-lir elements of the ciliary band, **d, e.** Transverse nerves of the body wall entering the ventral nerve cord in the middle of the ciliary band (**d**), and the posterior part of it (**e**). Abbreviations: *sc* – sensory cell, *vcb* – ventral ciliary band, *vnr* – varicoses of neurites, *vnc* – ventral nerve cord. Acetylated α-tubulin-lir – magenta, Octopamine-lir – blue, 5HT-lir – red, Substance P-lir – green. Stippled lines outline the borders of the ventral ciliary band

**Fig. 14:**
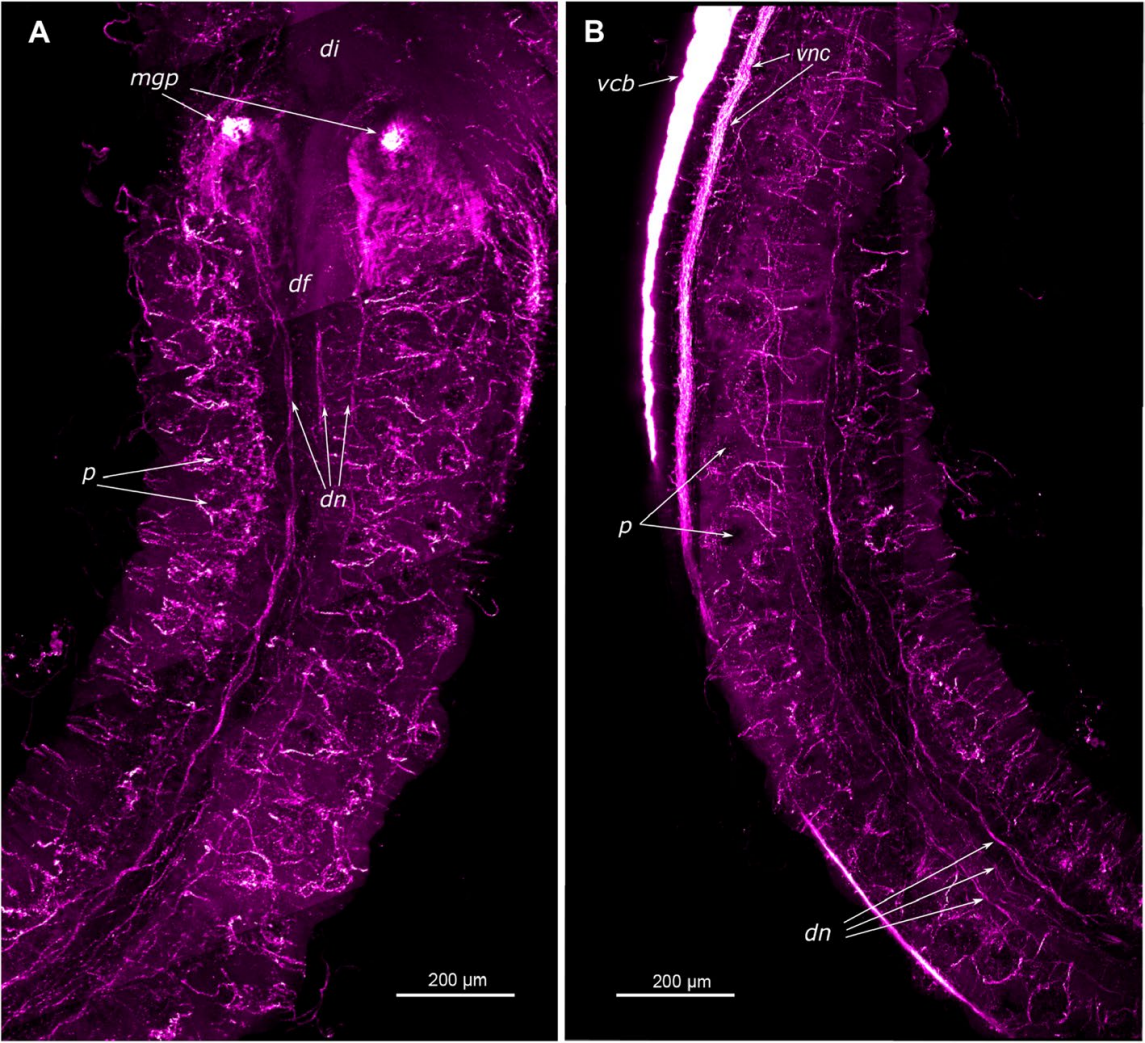
Innervation of genital papillae (**a**) and metameric area of the preannular region (**a, b**) visualized by immunolabeling against acetylated α-tubulin (magenta). Three loose dorsal longitudinal nerves (*dn*) can be distinguished. Note the dense nerve plexus, surrounding the papillae. Abbreviations: *di* – diaphragm, *df* – dorsal furrow, *dn* – dorsal nerve, *mgp* – male genital papillae, *p* – papillae, *vcb* – ventral ciliary band, *vnc* – ventral nerve cord

Intraepithelial nerve plexuses of the forepart and the trunk proper are generally similar in organization and have a predominantly diffuse arrangement (Fig. 8 d; Fig. 10 а). Most of these cells are diffusely connected to the ventral nerve cords that diverge behind the bridle and merge at the end of the metameric area of the trunk immediately posterior to the ventral ciliary band to continue as a single cord to the end of the trunk (Fig. 10 b; Fig. 13 e; Additional Fig. 2 a, b, e, f). The exceptions are the nerve plexuses located in the area of the muscular diaphragm and girdles (Fig. 11 a, b; Fig. 12) consisting primarily of neurites arranged in a circular pattern. Moreover, the arrangement that looks like a series of small irregular transverse nerves was observed near the ventral ciliary band in the metameric area of the trunk (Fig. 13 d). Several thin parallel nerves were found along the dorsal furrow; these nerves continue from the forepart into the trunk (Fig. 14). A significant number of open-type cells (whose apices contact the epithelial surface) probably representing sensory cells, as well as uni- and bipolar neurons of the closed type (that do not reach the epithelial surface) are present in the nerve plexuses among the varicose nerve fibers. The neurites and perikarya showed immunoreactivity against acetylated α-tubulin, SP, FMRFamide, 5-HT, and probably against octopamine.

FMRFamide- and 5-HT-immunoreactivity is predominant in neurons, i.e. the cells that have no contact with the body surface. Acetylated α-tubulin immunoreactivity was present not only in the processes of these cells but also in the majority of their perikarya. FMRFamide- and 5-HT-immunoreactive neurites extend their branches into the basal region of the epithelium and the musculature (Fig. 8 d; Fig. 11 b, d; Fig. 12 a; Additional Fig. 1 d, e). Some neurons are immunoreactive to SP and a few, mostly bipolar, neurons stain positively for octopamine (Fig. 13 a, c).

Solitary sensory cells are scattered throughout the body epithelium of *O. haakonmosbiensis*. Most sensory cells have a triangular shape because of the tapered apical portion without a distinct dendrite (Fig. 10 b, e; Additional Fig. 3). Only a small proportion of sensory cells are morphologically more differentiated, having a bipolar shape because of the formation of the cell body and dendrite (Fig. 10 d). Anti-tubulin antibody revealed single cilia on the apical surface of most sensory cells. Some cells have a short cilium, probably no longer than 2 μm, while others have a longer cilium 8–10 μm in length (Fig. 10 с). The cilia of these cells are not distinguishable by scanning electron microscopy. In addition to acetylated α-tubulin immunoreactivity, most sensory cells in the epithelium of the trunk stain positively for octopamine (Fig. 10 b), and a few sensory cells are immunoreactive to SP (Fig. 10 d). The axons of sensory cells form an intraepithelial nerve plexus and extend into the ventral nerve cords.

#### Ventral ciliary band

A considerable number of SP-immunoreactive sensory cells, a small number of 5-HT-immunoreactive sensory cells (Fig. 11 c–e; Fig. 13 a, b, Additional Fig. 2 c, d), and some sensory cells showing positive staining against octopamine (Fig. 13 с) are present in the epithelium among the ciliated cells along the entire length of the ciliary band in the metameric area of the trunk. All these cells are similar in morphology to the epithelial sensory cells of the trunk described above, but most cells in the ciliary band bear a single cilium about 4 μm in length. The length of the cilia cannot be measured accurately due to the intensive fluorescence of numerous long cilia of the surrounding epithelial cells. Sensory cells in the ciliary band are more densely distributed than in the trunk epithelium and are dominated by SP-immunoreactive cells. FMRFamide-, 5-HT-, and SP-immunoreactive neurons are located near the nerve cords on each side of the ciliary band. Most of these neurons show positive reaction to 5-HT (Fig. 11 d). An extensive diffuse nerve plexus is present at the base of the epithelium of the ciliary band; this plexus probably consists of axons from CNS and axons of the sensory cells revealed in the epithelium. Quite a few of these neurites are immunoreactive to 5-HT, octopamine, and SP and form varicosities at the base of the ciliated epithelial cells (Fig. 11 c, d; Fig. 13 a, c). The subsidiary ciliary field in the nonmetameric area of the trunk contains similar nerve elements, but sensory cells are significantly less numerous.

#### Papillae

**Fig. 15:**
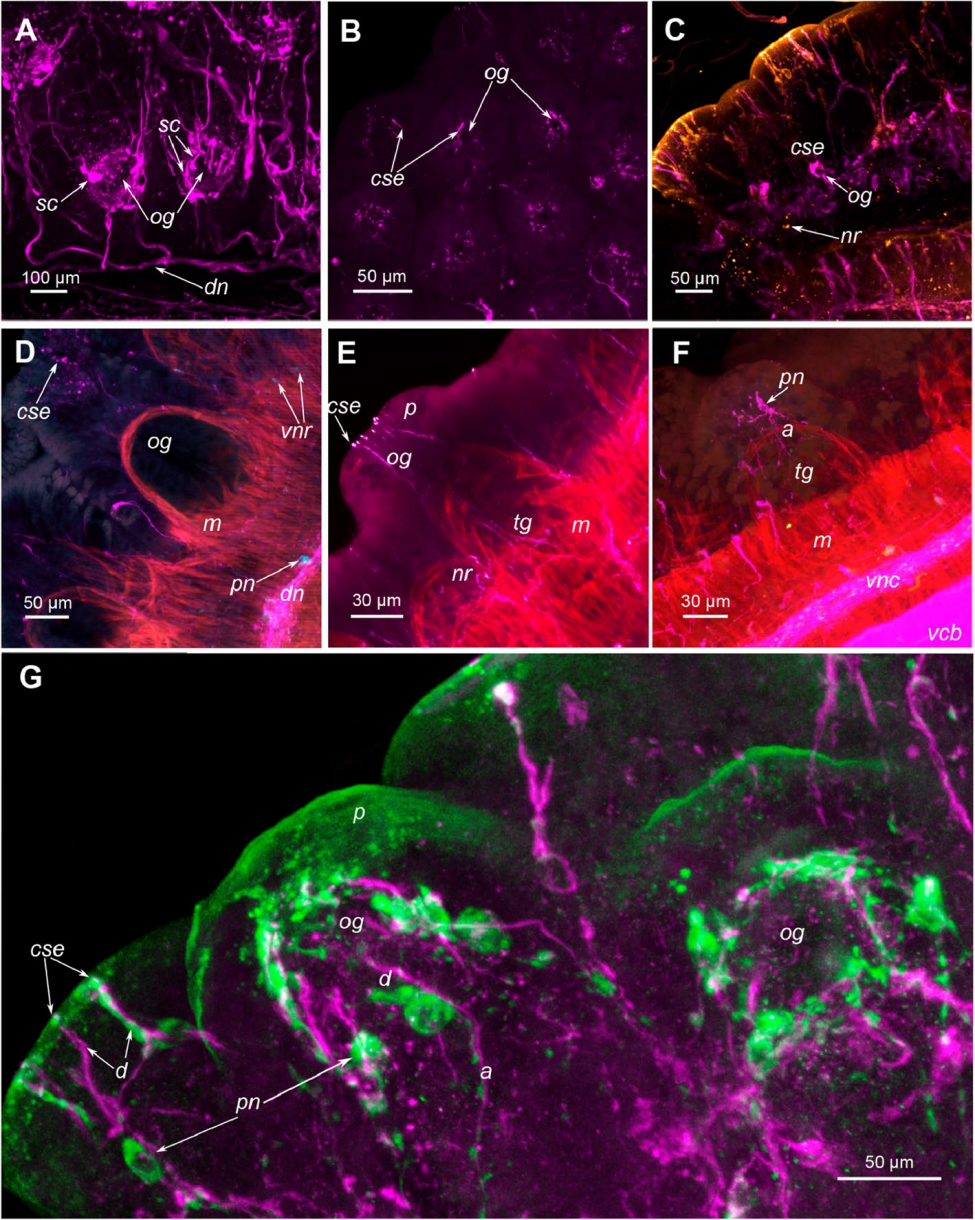
Innervation and musculature of papillae of the metameric area. **a, b.** Successive partial Z-projections showing general innervation of papillae (**a**), and sensory cilia surrounding ducts of the tubiparous glands (**b**). **c.** 5-HT-lir elements of papillae, **d, e, f.** Partial Z-projections showing details of acetylated α-tubulin-lir innervation and musculature of papilla and tubiparous glands. **G.** SP-lir sensory elements surrounding ducts of tubiparous glands of the papillae. Abbreviations: *a* – axon, *cse* – monociliated sensory ending, *d* – dendrite, *dn* – dorsal nerve, *m* – musculature, *og* – opening of the tubiparous gland duct, *sc* – sensory cell, *p* – papillae, *pn* – perikarya, *vnr* – varicose neurites, *vcb* – ventral ciliary band, *vnc* – ventral nerve cord, *nr* – neurites, *tg* – tubiparous gland. Muscles – red (phalloidin staining), acetylated α-tubulin-lir – magenta, FMRFamide-lir – cyan, 5HT-lir – orange, Substance P-lir – green

Papillae are present over the entire surface of the trunk of *O. haakonmosbiensis*. Their arrangement is closely associated with duct openings of subepithelial tubiparous glands. The neuronal organization of the papillae varies somewhat in different parts of the body, but these differences are caused primarily by the position of the openings of gland ducts on the papillae and the density of the papillae. The most densely distributed papillae are in the metameric area of the trunk. Duct openings in this body region are located closer to the apices of the papillae near their cuticular plaques (Fig. 3 b). These papillae are innervated by the dorsal nerves passing between the rows of papillae in the dorsal furrow (Fig. 14; Fig. 15 а). No evidence was found for innervation of the papillae by the ventral nerve cords. The ducts of the tubiparous glands occupy a significant portion of these papillae and each gland has its own intrinsic musculature. The glands are associated with the muscle fibers that spiral around the distal secretory parts of the glands and the ducts (Fig. 15 d–f). The duct opening on each papilla is surrounded by monociliary sensory cells lying in the epithelium around the tubiparous gland (Fig. 15 b). The cilia of these cells are clearly distinguishable on the body surface (Fig. 3 e). A major part of sensory cells is immunopositive for acetylated α-tubulin and SP (Fig. 15 g), but some of them are labeled by anti-tubulin antibody only. Each papilla together with the associated gland is abundantly innervated (Fig. 14; Fig. 15 a), but only a few of these neurites are immunoreactive to 5-HT and FMRFamide (Fig. 15 c, d). The duct of the tubiparous gland is surrounded by its own plexus located in the epithelium of each papilla below sensory cells; this plexus contains various neurites including varicose branching axons of sensory cells (Fig. 15 g).

**Fig. 16:**
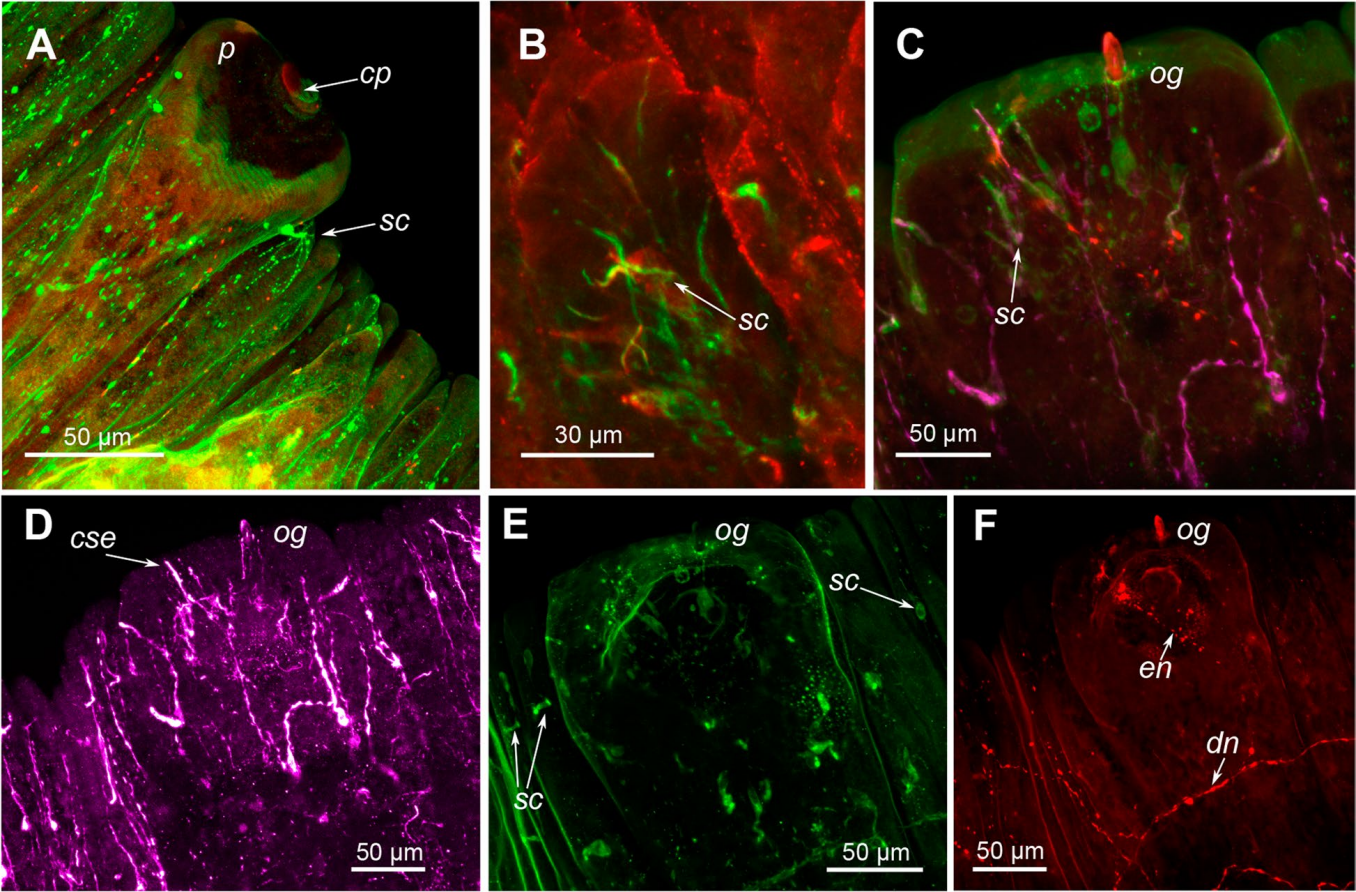
Innervation of papillae of the nonmetameric region. **a.** General view on the body wall with papilla. **b.** SP-lir sensory cells contributing to the plexus of papilla. **c, d, e, f.** Organization of SP-lir (**c**, **e**), acetylated α-tubulin-lir (**d**), and 5HT-lir (**c**, **f**) nerve elements of the papillae. Abbreviations:* cp* – cuticular plate, *cse* – monociliated sensory ending, *dn* – dorsal nerve, *en* – efferent neurites, *og* – opening of the tubiparous gland duct, *p* – papilla, *sc* – sensory cell. Acetylated α-tubulin-lir – magenta, 5HT-lir – red, Substance P-lir – green

Small papillae of the nonmetameric area of the trunk have a generally similar neuronal organization but are innervated by a smaller number of neurites. These papillae show a larger number of 5-НТ-positive elements, forming in some cases free nerve endings beneath the cuticular plaque of the papilla (Fig. 16 c, f). Ducts of the tubiparous glands open at the apices of these papillae near the cuticular plaque. The epithelium of the papillae also contains monociliary sensory cells that are positive against acetylated α-tubulin and SP (Fig. 16 a–e). Similar solitary sensory cells and sensory endings are also encountered in the epithelium surrounding the papillae (Fig. 16 a, c–e).

**Fig. 17:**
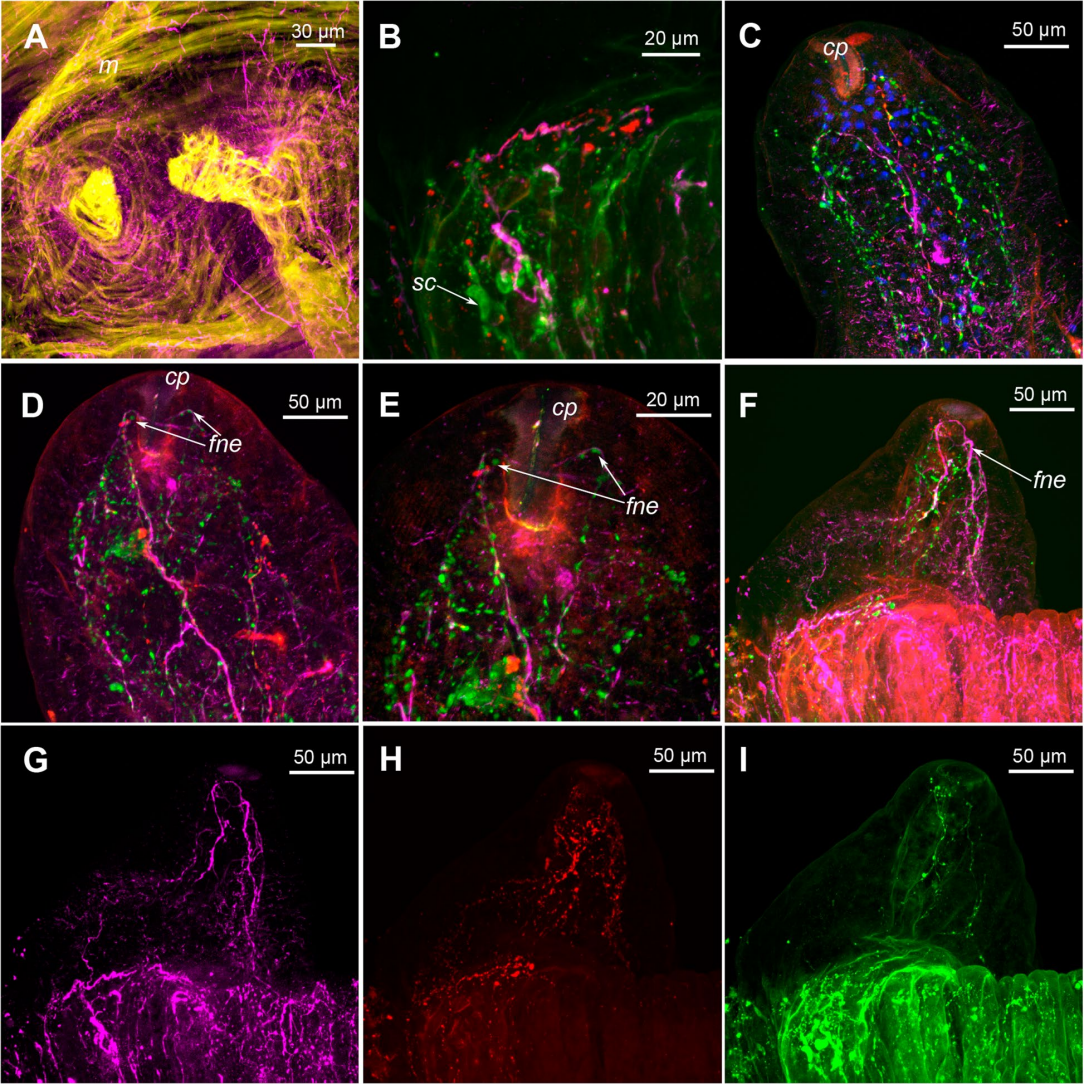
Musculature and innervation of enlarged papillae. **a**. Musculature and innervation revealed by anti-acetylated α-tubulin antibody. **b**. Body wall adjacent to the enlarged papilla. **c-f**. The organization of SP-lir (green) and 5HT-lir (red) innervation of enlarged papillae. Cell nuclei (blue) on **c** are counterstained by DAPI. **e**. The tip of papilla at higher magnification. Note the nerves underlying the cuticular plate. **g-i.** Separated fluorescent channels showing acetylated α-tubulin (**g**), 5-HT (**h**), and. SP-lir (**i**) nerve elements of the same papilla. Abbreviations: *cp* – cuticular plate, *fne* – free nerve endings, *m* – musculature of the papilla. Muscles – yellow (phalloidin staining), acetylated α-tubulin-lir – magenta, 5HT-lir – red, Substance P-lir – green

Enlarged papillae located above the second portion of the ventral ciliary band are innervated almost as abundantly as the large papillae in the metameric area of the trunk and possess a well-developed musculature (Fig. 17 a, f). These papillae are innervated by neurites that show immunoreactivity to acetylated α-tubulin, 5-HT, FMRFamide, and SP, and have their own intrinsic neuropil containing an array of neurites studded with varicosities and terminals. The papillae are supplied predominantly by 5-HT-immunoreactive neurites. Some of the 5-HT- and SP-immunoreactive neurites form free nerve endings immediately beneath the cuticular plaque, which can be mechanoreceptive terminals (Fig. 17 c–e). Immunostaining against acetylated α-tubulin and SP, however, revealed a much smaller number of monociliary sensory cells in these papillae, only 1–3 cells per papilla (Fig. 17 b). This could be explained by the observation that most sensory cells are located near the duct openings of the tubiparous glands located at the base of the papillae, rather than at their apices.

## Discussion

**Fig. 18:**
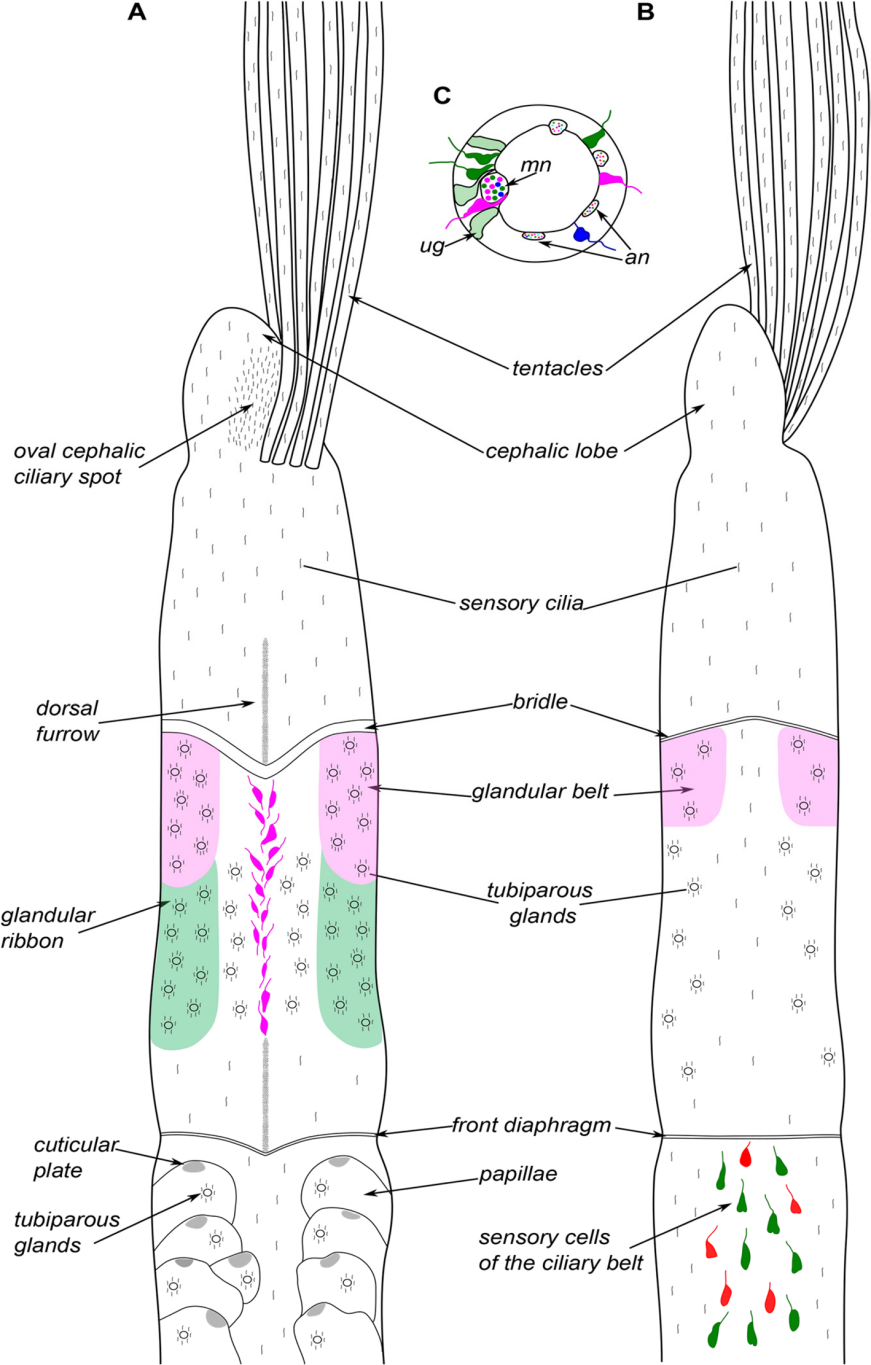
Generalized schematic drawing of *Oligobrachia haakonmosbiensis* receptor elements arrangement through the body. **a.** dorsal view, **b.** ventral view, **c.** cross-section through the tentacle. Abbreviations: *an* – additional tentacular nerves, *mn* – main tentacular nerve, *ug* – unicellular glands. Acetylated α-tubulin-lir – magenta, FMRFamide-lir – cyan, Octopamine-lir – blue, 5HT-lir – red, Substance P-lir – green

**Fig. 19:**
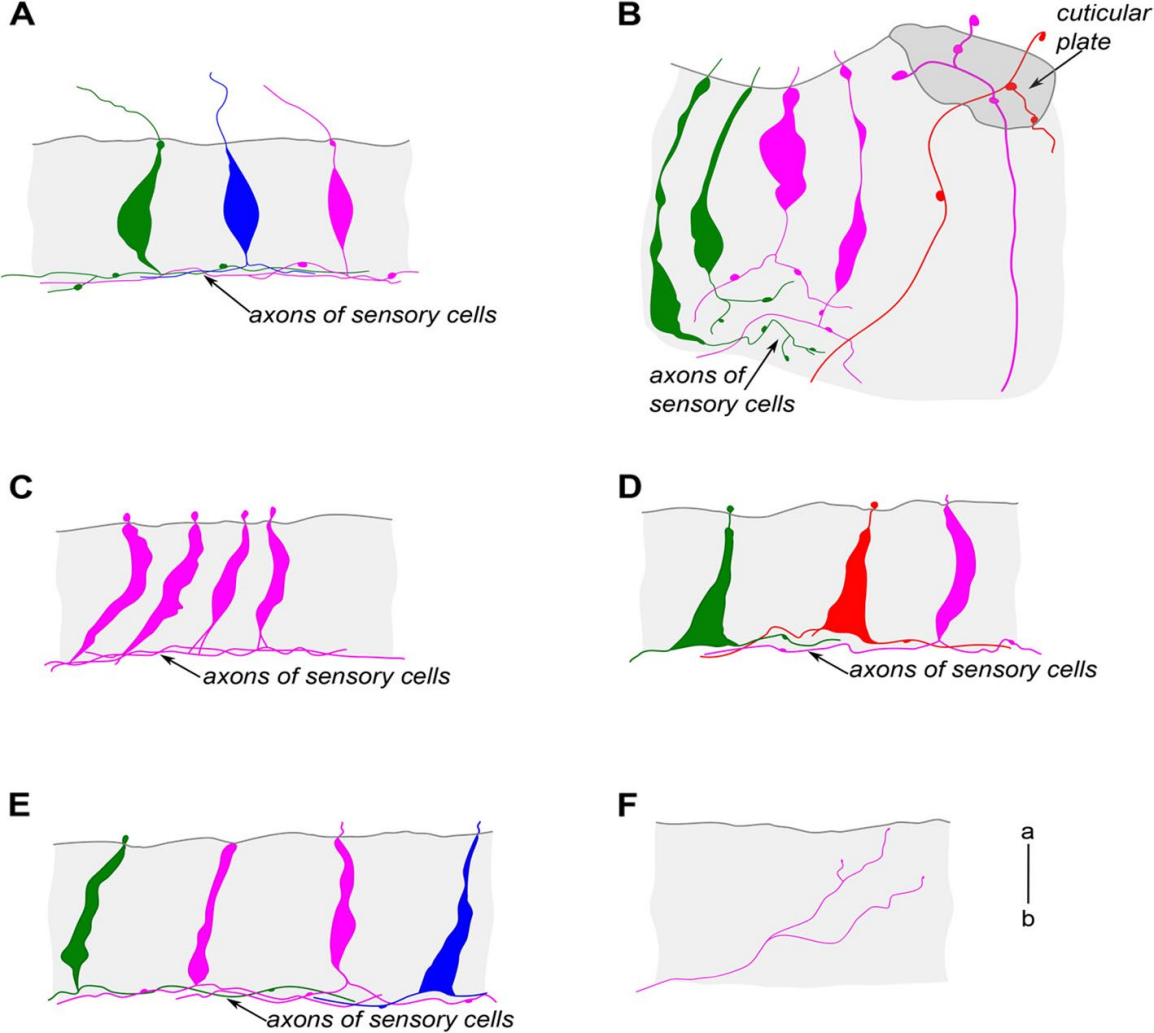
A variety of *Oligobrachia haakonmosbiensis* receptor cells and their processes in different body regions representing their distribution, chemical specificity, and cell shape. **a.** Tentacles. **b.** Papillae and tubiparous glands. **c.** Dorsal furrow. **d.** Ventral ciliary band. **e, f.** Body wall epithelium. Acetylated α-tubulin-lir – magenta, Octopamine-lir – blue, 5HT-lir – red, Substance P-lir – green. a-b line labels the direction of the apical-basal axis

**Fig. 20:**
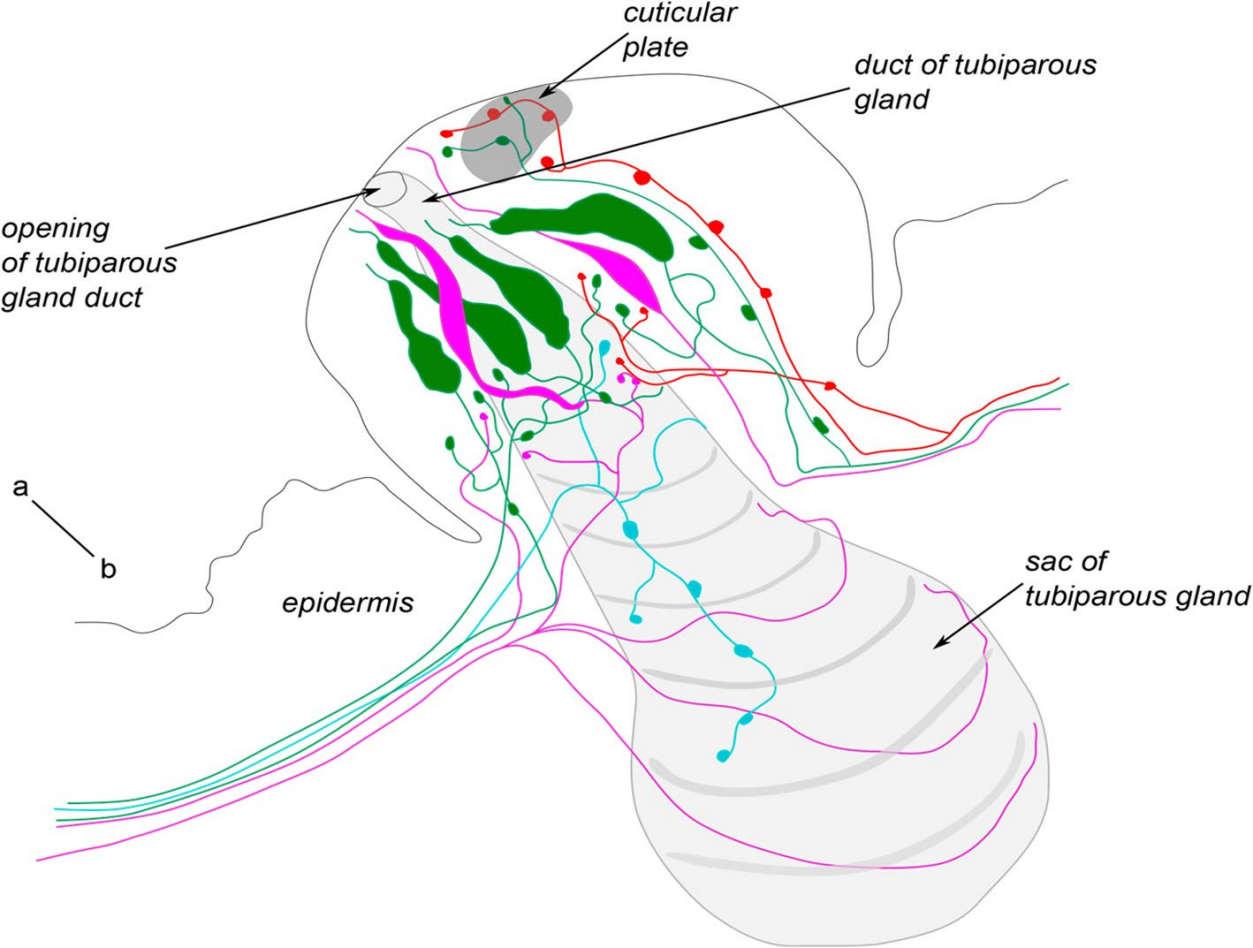
Schematic drawing of *Oligobrachia haakonmosbiensis* papilla innervation showing distribution of receptor and effector elements as well as concentrations of terminal branches of their processes around the duct of the tubiparous gland. Acetylated α-tubulin-lir – magenta, FMRFamide-lir – cyan, 5HT-lir – red, Substance P-lir – green. a-b line labels the direction of the apical-basal axis

In the present study we have shown that pogonophorans possess numerous and various sensory structures (Figs. 18, 19, and 20). These structures comprise both solitary primary sensory cells scattered in the epidermis throughout the body and the sensory systems that could be regarded as specialized sensory organs: cephalic tentacles, an oval ciliary spot on the cephalic lobe, the dorsal furrow, and numerous assemblages of papillae and tubiparous glands. Annelids are well known to exhibit a great variety of sensory organs [19, 46]. The principal and most common sensory systems are palps and other tentacular structures of the head region, the eyes, the photoreceptor-like sense organs, statocysts, nuchal organs, and sensory structures of the pygidium [19, 46–53]. In some studies, a great number of sensory cells have been shown to be present outside the sensory organs throughout the body wall of annelids [54, 55].

In all annelids studied to date, regardless of their lifestyle and phylogenetic position, transmission and scanning electron microscopy have revealed both poly- and monociliary receptor cells [19, 45–47, 52, 53, 55–59]. Some sensory cells have been shown to either lack cilia or have the cilia that do not project beyond the cuticle [46]. Our study has shown that all ciliary sensory cells found in *O. haakonmosbiensis* are monociliary, although the lengths of their cilia can be different. No polyciliary cells have been found, but some cells lacked cilia and some presumable free nerve endings also appeared to be present (Fig. 19). It should be noted that monociliary sensory endings have also been found on the body surface of the larvae of *O. haakonmosbiensis*.

The ciliary pit found at the dorsal side of the *O. haakonmosbiensis* trochophore may correspond to the dorsal sensory organ reported for the trochophore of the errant annelid *Phyllodoce maculata* [60]. This unpaired posterior sensory organ is situated at the dorsal midline of the hyposphere and consists of five bipolar sensory cells. Each cell bears an apical dendrite with a single cilium. The authors suggested that posterior sensory organ may perform mechano- or chemosensory functions. In *O. haakonmosbiensis* larvae the posterior ciliated pit possesses a similar number of cilia and is located also at the dorsal midline below the prototroch. Similar “dorsal ciliary spot” was also reported for *Siboglinum fiordicum* larvae [61]. Unfortunately, to date there is neither data about the cellular organization of this ciliated organ in pogonophorans nor information about such sensory organs in other annelid or molluscan larvae, but it seems highly probable that the dorsal ciliary pit of pogonophoran trochophores may perform the same functions as the dorsal sensory organ of phyllodocid larvae.

Although there is a large body of histochemical and immunohistochemical studies of the annelid nervous system, the focus in most of them has been on the CNS, while the peripheral nervous system has received little attention [62, 63]. The chemical modality of sensory cells in annelids has been examined in only a few studies. In the polychaete *Ophryotrocha puerilis,* a considerable number of catecholamine-reactive primary bipolar sensory cells have been revealed in the body appendages (antennae, palps, urites, and parapodial cirri), in the body wall, and the oesophageal wall using glyoxylic acid induced fluorescence (GIF) and immunohistochemistry [54, 55]. Some of these cells have been demonstrated to be mono- or polyciliary. Catecholamine-reactive sensory cells in annelids, nemerteans, and gastropods have been suggested to have a mechanosensory function [54, 55, 64, 65]. Electrophysiological methods have shown that the catecholamine-reactive sensory cells participate in the regulation of locomotion in the earthworm *Lumbricus terresrris* [66]. There is also evidence that some bipolar 5-HT immunoreactive cells have a possible sensory function in annelids [62, 63]. Nevertheless, the function of many sensory structures found in annelids is still uncertain or completely unknown.

The present study has shown that acetylated α-tubulin antibody labeling reveals a number of sensory cells and neurons in the peripheral nervous system. However, only a relatively small subset of these cells is immunopositive for FMRFamide, SP, 5-HT, or octopamine, which indicates that the pogonophorans nervous system has far more regulatory substances besides these chemicals. The presence of a huge number of neuropeptides was also shown for another annelid, *Platynereis dumerilii* [67]. Some of them were found to be annelid- or species-specific. This may also be true for *O. haakonmosbiensis*, and one can expect to find in siboglinids a large repertoire of neuropeptides that would be of extreme importance to answer the question of annelid nervous system plasticity and evolution.

Many of the detected primary sensory cells are immunopositive for SP (Fig. 19). These predominantly monociliary sensory cells are abundantly present in the cephalic tentacles, in the ciliary band, in the papillae, and around the duct openings of the tubiparous glands. Solitary SP-immunopositive receptor cells are also scattered throughout the epidermis. The arrangement of these cells, their number, and the presence of rigid cilia indicate that they might be mechanosensory. The secretion of the tubiparous glands is known to be used as a building material for the tubes in which the pogonophorans live [5, 37]. The ubiquitous presence of SP-immunopositive receptor cells around the duct openings of the tubiparous glands can be associated with the need for mechanosensory response to confirm the contact of the duct opening with the surface of the tube, which is followed by the discharge of secretion and its subsequent spreading over the inner surface of the tube by the papillae. The axons of the sensory cells encircling the duct openings of the tubiparous glands and the nerve fibers that innervate the musculature of the glands form plexus-like structures (Fig. 20). This raises the possibility that the glands are controlled by the local reflex arcs triggering the discharge of secretion from the tubiparous gland in response to tactile stimulation of the sensory cilia surrounding the duct pore. It should be noted that there is evidence in the literature indicating that in vestimentiferans single cilia can be present near some pores of the pyriform glands that are analogous to the tubiparous glands of the pogonophorans [33]. However, the papillae of vestimentiferans are not associated with tube-secreting glands [26, 32, 68]. These findings suggest that the tube-building mechanisms including the system of secretion and distribution of the tube material are different in vestimentiferans and pogonophorans. Tubiparous glands of vestimentiferans are situated primarily on the vestimental wings and it is these wings rather than papillae that are used by the worms when they build the walls and funnels of the tube [26, 32, 68]. The papillae are also lacking in the region of the pogonophoran body that may correspond to the vestimentum (see, however, the discussion on the homology of different body regions in pogonophorans, e.g.: [8, 31, 36]), but multicellular tubiparous glands are present in all frenulate pogonophorans, and the bridle and epidermal folds in the representatives of the genus *Galathealinum* Kirkegaard, 1956 can be regarded as a functional counterpart of the vestimental wings [69].

In addition to the participation in tube building, the papillae of the pogonophorans, like those of the vestimentiferans, help the animals to move inside the tubes and attach to their walls [26, 37]. The apices of the papillae bear cuticular plaques. These plaques together with the bridle and chaetae are thought to increase the worm’s purchase on the tube wall [5, 37], which implies the presence of tactile sensitivity when these structures contact with the wall of the tube. Free nerve endings that have been found in this study beneath the cuticular plaques on the papillae of *O. haakonmosbiensis* can be involved in this process (Fig. 19; Fig. 20). Sensory endings that have the morphology of free nerve endings are also present in the epithelium throughout the worm’s body. They are immunoreactive for antibody staining against acetylated α-tubulin, 5-HT, and SP. It is noteworthy that the vestimentiferans also have sensory structures in the papillae that can be revealed using the Golgi-Colonnier silver impregnation technique (our unpublished data).

Serotonin-immunoreactive receptor cells in *O. haakonmosbiensis* have only been observed in small numbers*,* in the epithelium of both ventral ciliary fields. In all other regions of the trunk and forepart, 5HT-positive elements were represented for the most part either by various neurites or by perikarya of uni- or bipolar neurons. These results are in good agreement with the observations made for other annelids, in which the bodies of 5HT-positive cells have been revealed predominantly in the CNS, while the peripheral regions of the nervous system have been shown to be occupied primarily by the processes of these cells [62, 63, 70, 71]. In a wide range of invertebrates, serotonin is involved in the regulation of ciliary activity [72]. Judging from the distribution of 5HT-positive neurites, it can be assumed that in the peripheral nervous system of *O. haakonmosbiensis* serotonin participates primarily in the regulation of ciliary cells, glandular structures, and to a lesser extent the musculature.

The presence of three ciliary bands in *O. haakonmosbiensis* including a subsidiary one that consists of several ciliary cells located some distance from the main ciliary band is very important. This fact lends further support to the idea that intercalary growth is the principal modus operandi for the formation of not only subsidiary ciliary bands, but also of all main regions and parts of the trunk [73]. The same mechanism could be involved in the processes by which the girdles of toothed chaetae are increased in number, the body is elongated between the transverse rows of papillae in the postannular region of the trunk and second-order metameres of the papillae appear in the metameric and nonmetameric areas of the trunk [74, 75].

Only a few putative octopamine-reactive cells have been found in *O. haakonmosbiensis*. All of them are monociliary cells that are encountered in the cephalic tentacles and sometimes in the epithelium of the body wall. It is possible that the presence of only a small number of cells could be explained by inconvenient conditions of fixation (4%PFA instead of 4%PFA with 0.3% glutaraldehyde, recommended by the antibody manufacturer) and/or by the fact that the predominant catecholamine in lophotrochozoans is dopamine, rather than octopamine [54, 55, 64, 65].

The presence of FMRFamide-immunoreactive sensory cells has not been unambiguously confirmed. FMRFamide-immunoreactivity was shown primarily for numerous uni- and bipolar neurons in the epithelial plexuses and within the ventral nerve cords. In the peripheral nervous system of *O. haakonmosbiensis,* FMRFamide is likely to perform predominantly efferent functions and may participate in the regulation of the musculature of the trunk, tentacles, bridle, diaphragm, girdles, papillae, and tubiparous glands.

Apart from the complexes of tubiparous glands and papillae, the highest concentration of sensory cells in *O. haakonmosbiensis* has been found in the epithelium of the cephalic tentacles, in the area of the anterior portion of the dorsal furrow, and the ventral ciliary band (Fig. 18). The tentacles of *O. haakonmosbiensis* have innervation and arrangement of sensory elements similar to those of the tentacles and palps of some other annelids. The palps of Spionidae and Protodrilidae, for example, bear numerous receptor cells, including presumably chemo- and mechanoreceptive elements, and most of them are arranged in rows above the corresponding nerves [47, 76–78]. Thus, it is highly possible that the cephalic tentacles of the pogonophorans contain chemo- and mechanoreceptive cells. In *O. haakonmosbiensis,* only some of the sensory cells revealed with anti-acetylated α-tubulin antibody staining are immunopositive for SP or octopamine, and a significant portion of cells have unknown chemistry. The main nerve is mostly sensory, but a few receptor cells are also arranged along the other nerves (Fig. 18). It is likely that the remaining nerves contain predominantly effector neurites (including those that are immunoreactive for 5-HT and FMRFamide) that form terminal branches in the musculature and on the glandular cells of the tentacles, which entails their participation in the regulation of the musculature, unicellular glands, and epithelial cells.

The organization of the dorsal furrow nerve plexus is of special interest. This area contains distinct dorsal nerves. Numerous intraepithelial receptor cells each carrying a very short cilium are arranged along the length of the nerves. These nerves, in turn, are associated with numerous multicellular and unicellular glands, and with the papillae in the trunk (Fig. 18). In the forepart behind the bridle, the receptor cells are especially abundant, and innervation of the glands is particularly extensive. This area contains two specialized paired glandular structures that are compact clusters of unicellular intraepithelial glandular cells interspersed with ducts of tubiparous glands. These glandular structures have previously been described as circular belts and longitudinal bands in unstained whole-mount preparations of many pogonophoran species including *O. haakonmosbiensis*, but their organization has not been described and no assumption has been made as to their function [5, 28, 37, 44, 79, 80]. Our results have shown that these glandular structures are abundantly innervated by neurites of the dorsal nerves. Neurites are especially abundant at the bases of the secretory cells that are situated immediately behind the bridle and show non-specific fluorescence under the standard settings for the Alexa Fluor 633 fluorochrome (Fig. 18). These neurites are arranged in such a way that it is impossible to determine whether they are the processes of other cells that run toward the secretory cells or originate from the secretory cells themselves. In the latter case, it cannot be ruled out that these glands are neurosecretory, especially since secretory granules are also present in the basal portions of the cells. It is also possible that these secretory cells are endocrine glands performing regulatory functions by paracrine secretion. Since the glands are located in the vicinity of the male genital openings, it can also be assumed that their secretory products and receptor cells of the dorsal furrow are involved in the regulation of reproductive behavior, for example, in the synchronization of gamete release by males and females. These assumptions, however, need further investigation.

Additional longitudinal nerves, including the dorsal nerves, have previously been described in many annelids, in particular, in the vestimentiferan *Lamellibrachia satsuma,* the polychaete *Polygordius appendiculatus,* and some species of Protodrilidae [15, 33, 47, 52, 81]. Nevertheless, these findings are mostly fragmentary and the methods used provide little information on the possible presence of sensory cells along these nerves. Our observations, however, have shown that in the protodrilid *Lindrilus flavocapitatus* a series of intraepithelial ciliary sensory cells of uniform morphology are arranged in two rows along the dorsal nerves (our unpublished observations). These cells, their cilia and the axons in the nerves have been revealed by the GIF method and showed green-blue fluorescence typical for catecholamines. It can therefore be anticipated that clusters of sensory cells observed along the dorsal nerves in the pogonophorans might also be present in other annelids.

The oval ciliary spot with regularly arranged separate cilia that has been found on the cephalic lobe of *O. haakonmosbiensis* is likely to be a sensory structure. However, the used microscopy methods failed to reveal any perikarya from which these cilia could originate. The possible function of this structure remains uncertain. Topographically, this area of clustered cilia partly overlaps with the area of phaosomal photoreceptors that has been described in three species of pogonophorans: the adult individuals of *Siboglinum (Nereilinoides) fiordicum* and *Oligobrachia gracilis* and the larvae of *Siboglinum (Siboglinum) poseidoni* [43–45]. Similar photoreceptors have also been found in leeches and oligochaetes, as well as in Protodrilidae and some other polychaetes [82–84]. These structures consist of two clusters of photoreceptors situated on the dorso-lateral sides of the cephalic lobe above the tentacular bases. In many annelids, eyespots and various tentacles (antennae) are present in approximately the same area [46]. Unlike the usual paired photoreceptor structures, the oval ciliary spot of *O. haakonmosbiensis* is unpaired. It also cannot be analogous to the paired nuchal organs present in most annelids, as the oval ciliary spot occupies a different position than the nuchal organs.

Since the development of the photoreceptor area in adult pogonophorans is directly linked to the corresponding larval structures [45], we have focused our attention on the ciliary sensory elements in the larvae of *O. haakonmosbiensis*. However, no analogies have been identified. In larval pogonophorans, the receptor cells have been described on the episphere of the trochophore of *S.(S.) poseidoni,* in the area of the apical organ [45]. The larvae of the same species have been reported to have a dorsal tuft of cilia beneath the prototroch, which was associated with the opening of the common excretory canal of the paired larval protonephridia [45]. Judging by the position of these cilia, it is possible that the larvae of *O. haakonmosbiensis* could have the same ciliary structure (Fig. 6a, b). This structure can be presumed to be a specialized dorsal larval sensory organ associated with the nephridiopore. In the larvae of *O. haakonmosbiensis,* the apical organ lacks a central tuft of cilia and appears as a terminal bulge at the apical pole of the trochophore episphere. At the base of this bulge, however, are separate asymmetrically positioned cilia that may have a sensory function (Fig. 6 f). A similar morphology of the apical organ has been described in some vestimentiferan larvae, while the apical organ in the larvae of *S. (S.) poseidoni* has a typical morphology with a central tuft of cilia [45, 85].

The present study has supplemented previous descriptions of ciliary larval sensory structures in the pogonophorans by describing, for the first time, other monociliary sensory structures in the larvae of *O. haakonmosbiensis,* most notable of which are two symmetrically arranged cilia on the pygidium and solitary cilia on the larval hyposphere (Fig. 6 g). These results support a broad diversity of sensory structures and an early development of sensory functions in the pogonophoran larvae.

In conclusion, it should be pointed out that the study of the general organization of intraepithelial nerve plexuses in the body wall of *O. haakonmosbiensis* has shown that these plexuses are poorly differentiated, despite the presence of a considerable number of sensory structures. All plexuses are mostly diffuse and their neurites and neuronal perikarya do not form distinct circular and longitudinal nerves, except in a few areas. Most neurites enter the ventral nerve cords also in a diffuse manner. This organization of plexuses is not typically observed even in the annelids with a completely intraepithelial nervous system such as Oweniidae, Polygordiidae, Protodrilidae, etc. [52, 70, 81] and has so far been described only in species of Dinophilidae that have a clearly progenetic morphology [71, 86, 87]. Our results, therefore, may add further support to the hypothesis of the neotenic origin of the pogonophorans [45, 73]. On the other hand, if the possibility of the intraepithelial nervous system being a plesiomorphic condition in annelids is taken into consideration [16], it can be argued that the pogonophorans could have also retained certain primitive traits in the organization of the nerve plexuses in the peripheral nervous system. Finally, such a primitive state of the peripheral nervous system of *O. haakonmosbiensis* may be a result of extreme simplification due to sedentary life conditions and specific environments. A somehow similar process apparently took place in Protodriliformia, where the miniaturization led to the formation of a characteristic body adopted for interstitial life as well as simplification of the nervous system [11]. Further studies of the organization of the peripheral nerve plexuses and sensory structures in different annelid groups are strongly needed to clarify this question.

## Conclusions

The present study has demonstrated, for the first time, that the pogonophorans as exemplified by *Oligobrachia haakonmosbiensis* have numerous and diverse sensory structures. In adult animals, these structures comprise both diffusely arranged solitary primary sensory cells located in the epidermis throughout the body as well as the sensory systems that could be viewed as possible specialized sensory organs: cephalic tentacles, an oval ciliary spot on the cephalic lobe, the dorsal furrow and numerous assemblages of papillae and tubiparous glands. In the larvae of *O. haakonmosbiensis,* the presumable ciliary sensory endings are present not only at the base of the apical bulge on the episphere, but also below the prototroch on the body and on the pygidium. Annelids display a great diversity of sensory organs, but the sensory structures identified in the pogonophorans are quite unusual, which makes a comparison difficult. No structures have been found in the pogonophorans that could be comparable to the nuchal organs. A certain similarity in innervation and sensory elements was observed between the tentacles in the pogonophorans and the palps and other cerebral tentacular structures in other annelids. Sensory structures associated with the complexes of tubiparous glands and papillae are probably unique for the pogonophorans because their presumed mechanosensory function is likely to be related to the regulation of the worm’s anchorage inside the tube and the characteristics of the tube-building process. Sensory structures of the dorsal furrow are presumably engaged in the regulation of reproductive behavior because they are situated in the vicinity of the male genital openings and are closely associated with two specialized paired glandular structures described in the present study for the first time. All ciliary sensory cells found in the adult pogonophorans proved to be monociliary, which is unusual for annelids. Although the monociliary sensory cells do occur in annelids, the polyciliary cells are by far more common. Most of the revealed cells are immunopositive against acetylated α-tubulin. Only a relatively small subset of these cells is immunopositive for FMRFamide, SP, 5-HT, or octopamine, which implies that the actual range of neurotransmitters present in the pogonophorans is not limited to only these substances. A low level of morphological differentiation of the pogonophoran peripheral nervous system, which is evident in the diffuse organization of intraepithelial nerve plexuses in the body wall is not typical even for those annelids that have an intraepithelial nervous system. This may be considered as evidence in favor of the hypothesis of the neotenic origin of the pogonophorans [45, 73]. It also cannot be ruled out that the pogonophorans could have retained certain plesiomorphic traits in the organization of the peripheral plexuses. Alternatively, the primitive organization of the peripheral nervous system may be, at least partially, a result of extreme adaptation to a sessile lifestyle in specific habitats.

## Materials and methods

Twelve specimens of *O. haakonmosbiensis*, some with numerous larvae in the anterior parts of their tubes, were obtained from four localities in the Norwegian and Laptev Seas (for samples examined, see Table 1). Gravity and box cores were used to collect the material. The sediments everywhere were mainly mud, sometimes with clay and stones. On the Haakon Mosby Mud Volcano (HMMV), the Storegga Slide, and the Laptev Sea sites, *Oligobrachia* occurs in cold seep conditions [88–92]. For scanning electron microscopy (SEM) the material was fixed in 2,5% glutaraldehyde in either seawater or cacodylate buffer and then transferred to ethanol. For Immunohistochemistry the specimens were fixed in 4% paraformaldehyde in 0.1 M phosphate buffered saline (PBS), rinsed with PBS and stored in PBS with the addition of 0.05% NaN_3_. All material is deposited at the Zoological Institute of the Russian Academy of Sciences, St. Petersburg (catalogue number ZIN No. HN65).

**Table 1:** Samples of *Oligobrachia haakonmosbiensis* Smirnov, 2000

Research Vessel	Station	Site	Lat.	Long.	Depth, m	Date	No. of spec.
Akademik Mstislav Keldysh	5947	Laptev Sea	76°28′6»N	125°29′27»E	72.5	26 Aug. 2018	4 spec.
Akademik Mstislav Keldysh	5953	Laptev Sea	76°32′9″N	127°28′58″E	63	28 Aug. 2018	3 spec.
Pourqoi-Pas?	4	Norwegian Sea, Storegga Slide	64°45.27′N	4°58.88′E	745	22 May 2006	2 spec. With larvae
Pourqoi-Pas?	108	Norwegian Sea, Haakon Mosby Mud Volcano	72°00.33′N	14°42.76′E	1261	6 June 2006	3 spec. With larvae

The specimens were studied using both confocal laser scanning microscopy (CLSM) and scanning electron microscopy (SEM). For SEM the specimens were first cleaned by twice alternating incubations in 20% ethanol and 16% glycerol in ethanol (6–12 h for each incubation). All incubations were performed on a shaker. Mature specimens and larvae were then dehydrated in ascending ethanol and acetone series, critical-point dried, placed on stubs, and coated with platinum. SEM micrographs were performed on a Quanta 250 (FEI Company, The Netherlands) electron microscope.

For CLSM analysis, the specimens were washed in PBS with 0.1% Triton X-100 (PBT), preincubated in blocking buffer (BB; PBS with 1% Triton X-100, 1% BSA, and 0.05% NaN_3_) overnight at + 4 °C. For primary antibody incubation, different combinations of antibodies against acetylated α-tubulin (Sigma-Aldrich, St. Louis, USA, T-6793, monoclonal, produced in mouse), serotonin (Immunostar Hudson, WI, USA, 20079, polyclonal, produced in goat, or Immunostar, 20,080, polyclonal, produced in rabbit), FMRFamide (Immunostar, 20,091, polyclonal, produced in rabbit), and Substance P (Immunostar, 20,064, polyclonal, produced in rabbit) diluted 1:1000–1:2000 in BB were used. The incubation was carried out for 24 h at + 4 °C followed by three washes in PBT for 20 min each. The following incubation with secondary antibodies (AlexaFluor 633, AlexaFluor 647, Thermo Fisher Scientific, Waltham, USA or CF 633, Sigma-Aldrich) diluted 1:500–1:800 was performed overnight at + 4 °C. After immunolabelling, the specimens were stained with 1 μg/ml TRITC-conjugated phalloidin (Sigma, P1951) and 1 μg/ml DAPI (Karl Roth, Karlsruhe, Germany, 6843.1) in PBT for 2 h, mounted in Mowiol and examined using Leica TCS SPE or Leica TCS SP5 laser confocal microscopes (Leica Microsystems, Wetzlar, Germany). The Bitplane IMARIS, as well as FIJI software, were used for 3D visualization and the analysis of confocal stacks. Images were further processed with Adobe Photoshop CS2 to adjust the contrast and brightness. The schematic drawings were made in Inkscape.

## Supplementary Information

**Additional file 1: Fig. S1.** Details of neuronal elements distribution in tentacles (a,b), forepart (c), and anterior part of the ventral ciliary band (d,e) of *Oligobrachia haakonmosbiensis*. a, b. Separated fluorescent channels showing 5-HT-lir (a) and b. SP-lir (b) fibers of tentacular nerves. See Fig. 7 h for the composite image. c. 5-HT-lir elements of the forepart. For the full composite image see Fig. 8 d. d. FMRFamide-lir elements in the commissural nerve of the diaphragm and ventral nerve cord. For the full composite image see Fig. 11 b. e. FMRFamide-lir (cyan) and acetylated α-tubulin-lir (magenta) in the commissural nerve of the diaphragm and ventral ciliary band. See Fig. 11a for the full composite image. Abbreviations: *an* – additional tentacular nerves, *cnd* - commissural nerve of the diaphragm, *mn* – main tentacular nerve, *pn* – perikarya, *ug* – unicellular glands, *vcb* – ventral ciliary band, *vnc* – ventral nerve cord.

**Additional file 2: Fig. S2.** Innervation of the body wall at the area of the ciliary band. a, b. Successive partial Z-projections showing motile cilia of the ventral ciliary band (a) and acetylated α-tubulin-lir (magenta) elements underlying them (b). c, d. Separated fluorescent channels showing SP-lir (c) and 5HT-lir (d) elements underlying the ciliary band. See Fig. 13 a for the composite image. e, f. Acetylated α-tubulin-lir elements of the middle (e) and posterior (f) parts of the ventral ciliary band. See Fig. 13 d, e for partial Z-projections devoid upper layers with motile cilia. Abbreviations: *sc* – sensory cell, *vcb* – ventral ciliary band, *vnr* – varicoses of neurites, *vnc* – ventral nerve cord.

**Additional file 3: Fig. S3.** Some details of nerve plexus organization it the preannular region. a. Octopamine-lir sensory cells (white) of the body wall. b. Octopamine-lir cells (white) with higher magnification. For the full composite image see Fig. 10 b. c. Sensory cells in the nerve plexus of the body wall epithelium of the preannular region. Cell nuclei are counterstained by DAPI (light blue). See also Fig. 10 e for the same image without DAPI. Abbreviations: *a* – axon, *d* – dendrite, *dnp* – diffuse nerve plexus, *sce* – monociliated sensory ending, *sc* – sensory cell.

## Data Availability

The datasets analyzed during the current study are available from the corresponding author on reasonable request. All data needed are included in the paper.
