# Supplementary figures and images for: Sensory cells and the organization of the peripheral nervous system of the siboglinid Oligobrachia haakonmosbiensis Smirnov, 2000
Source: BMC Zool. 2022 Mar 29;7:16. doi: 10.1186/s40850-022-00114-z (PMC10127031; doi:10.1186/s40850-022-00114-z)

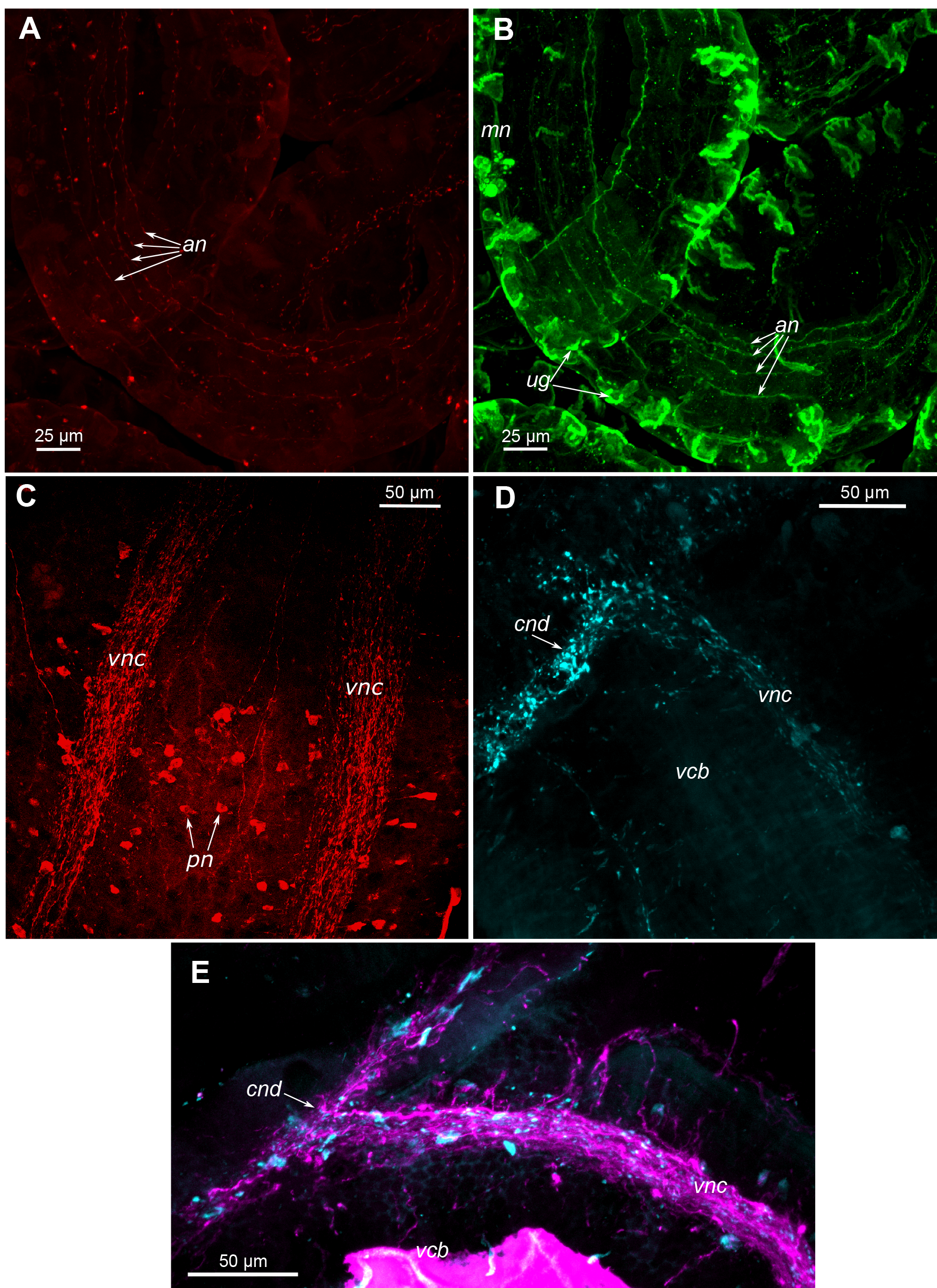

Supplement: Supplementary file 1 — Additional file 1: Fig. S1. Details of neuronal elements distribution in tentacles (a,b), forepart (c), and anterior part of the ventral ciliary band (d,e) of Oligobrachia haakonmosbiensis. a, b. Separated fluorescent channels showing 5-HT-lir (a) and b. SP-lir (b) fibers of tentacular nerves. See Fig. 7 h for the composite image. c. 5-HT-lir elements of the forepart. For the full composite image see Fig. 8 d. d. FMRFamide-lir elements in the commissural nerve of the diaphragm and ventral nerve cord. For the full composite image see Fig. 11 b. e. FMRFamide-lir (cyan) and acetylated α-tubulin-lir (magenta) in the commissural nerve of the diaphragm and ventral ciliary band. See Fig. 11a for the full composite image. Abbreviations: an – additional tentacular nerves, cnd - commissural nerve of the diaphragm, mn – main tentacular nerve, pn – perikarya, ug – unicellular glands, vcb – ventral ciliary band, vnc – ventral nerve cord. [file 40850_2022_114_MOESM1_ESM.png]

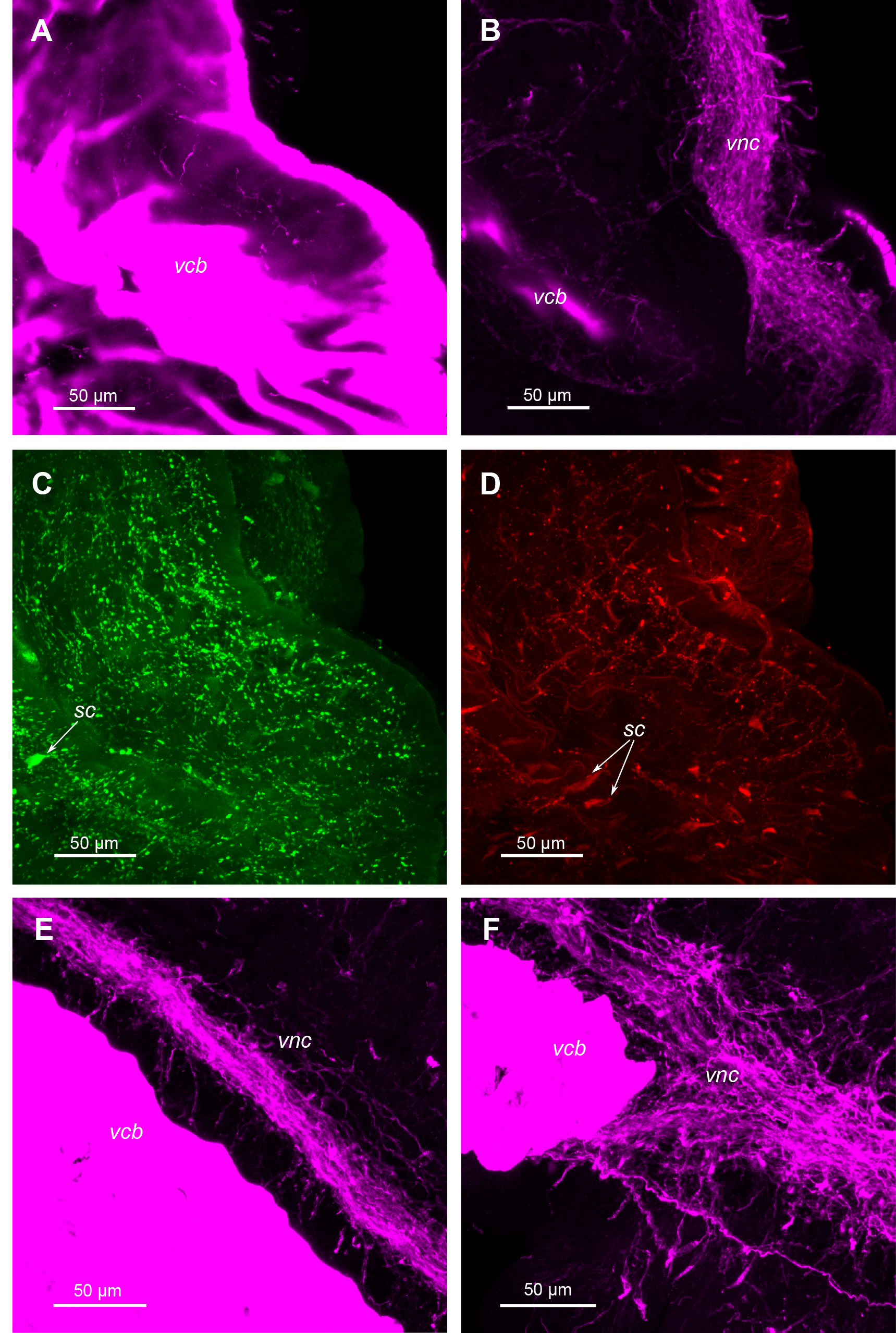

Supplement: Supplementary file 2 — Additional file 2: Fig. S2. Innervation of the body wall at the area of the ciliary band. a, b. Successive partial Z-projections showing motile cilia of the ventral ciliary band (a) and acetylated α-tubulin-lir (magenta) elements underlying them (b). c, d. Separated fluorescent channels showing SP-lir (c) and 5HT-lir (d) elements underlying the ciliary band. See Fig. 13 a for the composite image. e, f. Acetylated α-tubulin-lir elements of the middle (e) and posterior (f) parts of the ventral ciliary band. See Fig. 13 d, e for partial Z-projections devoid upper layers with motile cilia. Abbreviations: sc – sensory cell, vcb – ventral ciliary band, vnr – varicoses of neurites, vnc – ventral nerve cord. [file 40850_2022_114_MOESM2_ESM.png]

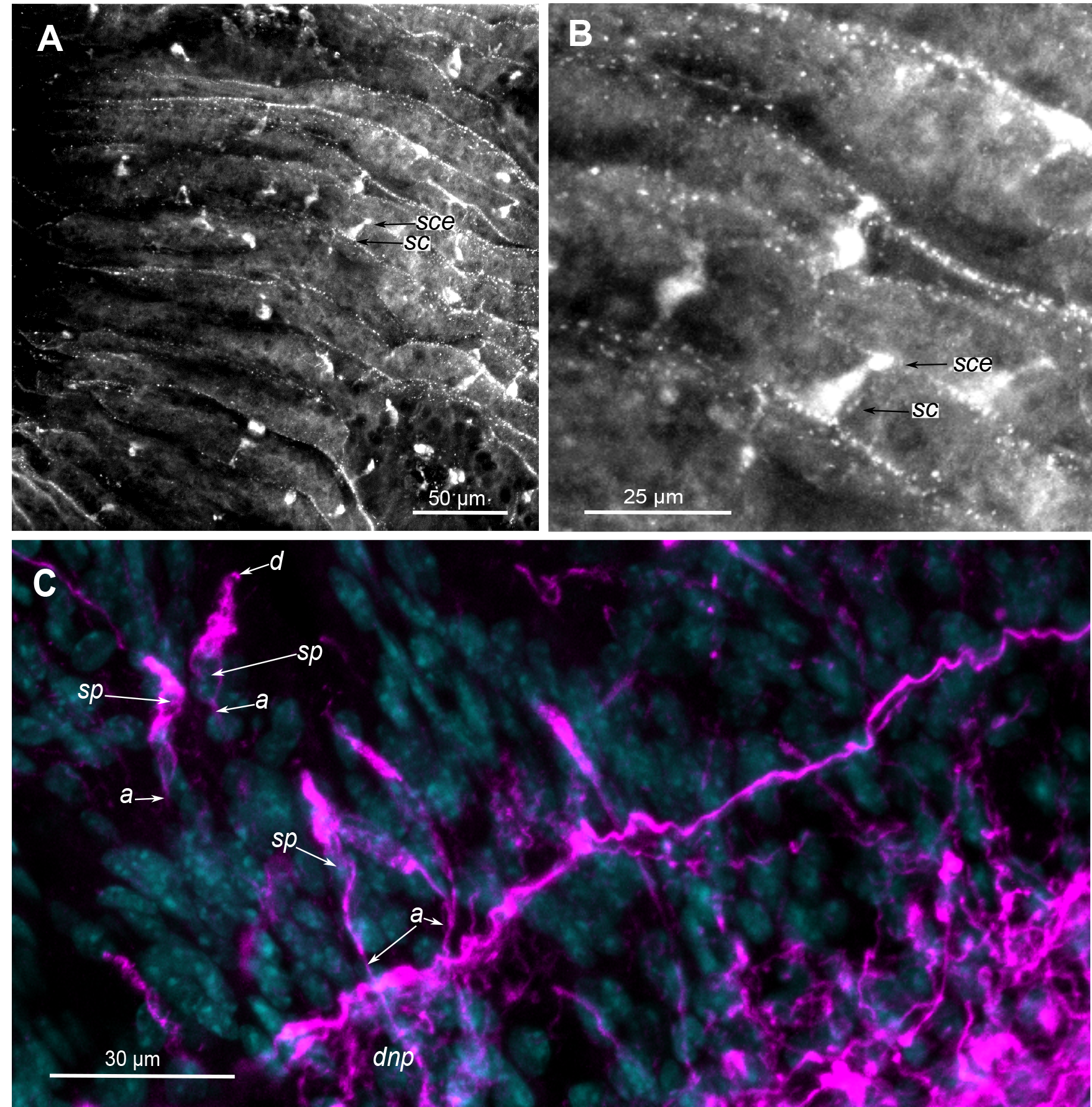

Supplement: Supplementary file 3 — Additional file 3: Fig. S3. Some details of nerve plexus organization it the preannular region. a. Octopamine-lir sensory cells (white) of the body wall. b. Octopamine-lir cells (white) with higher magnification. For the full composite image see Fig. 10 b. c. Sensory cells in the nerve plexus of the body wall epithelium of the preannular region. Cell nuclei are counterstained by DAPI (light blue). See also Fig. 10 e for the same image without DAPI. Abbreviations: a – axon, d – dendrite, dnp – diffuse nerve plexus, sce – monociliated sensory ending, sc – sensory cell. [file 40850_2022_114_MOESM3_ESM.png]
